# Long‐term changes to the frequency of occurrence of British moths are consistent with opposing and synergistic effects of climate and land‐use changes

**DOI:** 10.1111/1365-2664.12256

**Published:** 2014-04-29

**Authors:** Richard Fox, Tom H. Oliver, Colin Harrower, Mark S. Parsons, Chris D. Thomas, David B. Roy

**Affiliations:** ^1^ Butterfly Conservation Manor Yard Wareham Dorset BH20 5QP UK; ^2^ NERC Centre for Ecology & Hydrology Maclean Building Benson Lane Crowmarsh Gifford Wallingford, Oxfordshire OX10 8BB UK; ^3^ Department of Biology University of York York YO10 5DD UK

**Keywords:** citizen science, climate change, frequency of occurrence, habitat loss, invertebrate declines, land‐use change, Lepidoptera, moths

## Abstract

1. Species’ distributions are likely to be affected by a combination of environmental drivers. We used a data set of 11 million species occurrence records over the period 1970–2010 to assess changes in the frequency of occurrence of 673 macro‐moth species in Great Britain. Groups of species with different predicted sensitivities showed divergent trends, which we interpret in the context of land‐use and climatic changes.
2. A diversity of responses was revealed: 260 moth species declined significantly, whereas 160 increased significantly. Overall, frequencies of occurrence declined, mirroring trends in less species‐rich, yet more intensively studied taxa.
3. Geographically widespread species, which were predicted to be more sensitive to land use than to climate change, declined significantly in southern Britain, where the cover of urban and arable land has increased.
4. Moths associated with low nitrogen and open environments (based on their larval host plant characteristics) declined most strongly, which is also consistent with a land‐use change explanation.
5. Some moths that reach their northern (leading edge) range limit in southern Britain increased, whereas species restricted to northern Britain (trailing edge) declined significantly, consistent with a climate change explanation.
6. Not all species of a given type behaved similarly, suggesting that complex interactions between species’ attributes and different combinations of environmental drivers determine frequency of occurrence changes.
7. *Synthesis and applications*. Our findings are consistent with large‐scale responses to climatic and land‐use changes, with some species increasing and others decreasing. We suggest that land‐use change (e.g. habitat loss, nitrogen deposition) and climate change are both major drivers of moth biodiversity change, acting independently and in combination. Importantly, the diverse responses revealed in this species‐rich taxon show that multifaceted conservation strategies are needed to minimize negative biodiversity impacts of multiple environmental changes. We suggest that habitat protection, management and ecological restoration can mitigate combined impacts of land‐use change and climate change by providing environments that are suitable for existing populations and also enable species to shift their ranges.

## Abstract 2

Our findings are consistent with large‐scale responses to climatic and land‐use changes, with some species increasing and others decreasing. We suggest that land‐use change (e.g. habitat loss, nitrogen deposition) and climate change are both major drivers of moth biodiversity change, acting independently and in combination. Importantly, the diverse responses revealed in this species‐rich taxon show that multifaceted conservation strategies are needed to minimize negative biodiversity impacts of multiple environmental changes. We suggest that habitat protection, management and ecological restoration can mitigate combined impacts of land‐use change and climate change by providing environments that are suitable for existing populations and also enable species to shift their ranges.

## Introduction

The main drivers of global biodiversity change have been identified (Millennium Ecosystem Assessment 2005), but their impacts vary spatially, temporally and taxonomically. Drivers may also interact to produce synergistic or opposing effects (Travis 2003; Brook, Sodhi & Bradshaw 2008; Schweiger *et al*. 2010), but there are few empirical examples, particularly for insects, which comprise the majority of terrestrial biodiversity (Collen *et al*. 2012). Unquantified change and a resultant lack of evidence‐based conservation present pressing biological and strategic management challenges.

Here, we utilize a substantial data set of species occurrence records to examine long‐term changes in a species‐rich insect taxon (Lepidoptera: macro‐moths) in Great Britain (GB). Large‐scale, comprehensive assessments of biodiversity changes in speciose insect taxa are rare (Thomas 2005; Mattila *et al*. 2008, 2009; Jeppsson *et al*. 2010). Moths constitute one of the largest groups of herbivorous insects, forming key links in food webs, inflicting damage (as well as pollination) on their plant hosts and providing a major food source for insectivorous animals in many ecosystems (Strong, Lawton & Southwood 1984).

We calculate long‐term changes in frequency of occurrence of 673 lepidopteran species in GB and evaluate the trends in relation to species’ predicted sensitivities to recent climatic and habitat changes. Habitat modification, particularly agricultural intensification, is considered the pre‐eminent cause of recent species declines in GB and other western European countries (Warren & Key 1991; Robinson & Sutherland 2002; Kleijn *et al*. 2009). In parallel, climate change is eliciting changes in the geographical range, abundance, phenology and biotic interactions of Lepidoptera species (Parmesan 2006). Climate change provides a shifting context for the impacts of habitat modification, either amplifying or ameliorating species’ responses depending upon ecological traits and biogeographical situation.

Gradients of land use, climate and species’ distributions combine conveniently to provide distinct (often opposite) predictions of changes to species’ occurrence in GB. Northern GB retains a higher proportion of semi‐natural habitats than southern GB, where levels of land conversion to intensive agriculture and urbanisation have been greater (Morton *et al*. 2011). Therefore, moth species that are not strongly constrained by climate and occur widely in GB might be expected to decline in the south while remaining relatively stable in the north, in response to land‐use changes. On the other hand, many insect species (including many macro‐moths) reach the north‐western climatic limit of their European range within southern GB. These species should benefit from climate change, leading to the opposite prediction – they should potentially increase as the climate has warmed (Hickling *et al*. 2006). In contrast, arctic–alpine species that are restricted to northern and montane areas in GB might be expected to decline in response to regional warming. By considering warm‐adapted, cold‐adapted and relatively climate‐insensitive (within GB) species across a broad gradient of land‐use intensity, we attempt to tease apart the effects of change in land use and climate on GB moths.

Land‐use changes involve altered management (e.g. increased fertilizer input) as well as conversion from one land‐use type to another. We considered these effects by analysing the occurrence changes in moths that are monophagous on larval host plants that possess different environmental requirements. Trait‐based analyses of plant trends have been linked to drivers of change (Carey *et al*. 2008), utilizing Ellenberg indicator values to characterize the realized niches of plants along environmental gradients, such as those relating to soil chemistry and light availability (Ellenberg 1979). Thus, by considering the Ellenberg indicator values of moth larval hosts, we can examine links between drivers of botanical change and changes to the frequency of occurrence of moths.

Here, we test three hypotheses: (i) macro‐moth species will show a wide diversity of changes as they respond to diverse drivers, but will have declined overall, mirroring wider biodiversity trends. (ii) The responses of species with different geographical distributions (southern, northern, widespread) are expected to differ because the effects of climate and land use may differ between these species categories. (iii) Moth occurrence trends will be associated with host plant attributes (Ellenberg indicator values); specifically, moths that use types of plant that are in decline, such as those associated with low nitrogen soil conditions, will also be in decline.

We found support for each hypothesis, enabling us to assess long‐term moth biodiversity change. These results will guide future research into drivers of biodiversity change and inform ecological management to buffer species from negative impacts.

## Materials and methods

### Data sources

GB species occurrence records for macro‐moths (here defined as Lepidoptera families: Hepialidae, Cossidae, Zygaenidae, Limacodidae, Sesiidae, Lasiocampidae, Saturniidae, Endromidae, Drepanidae, Geometridae, Sphingidae, Notodontidae, Erebidae, Nolidae and Noctuidae) for the period 1970–2010 were obtained from the National Moth Recording Scheme data base: 11 074 870 records were extracted. These were collated from volunteer observers during recording for distribution atlases organized by the Biological Records Centre and Butterfly Conservation (Heath & Emmet 1983; Hill *et al*. 2010) (accessible via the National Biodiversity Network http://data.nbn.org.uk).

Interspecies detectability differences can be an issue with analysis of occurrence data (MacKenzie *et al*. 2006; Kéry, Gardner & Monnerat 2010), so we only considered within‐species changes over time. New knowledge of species’ biology or novel collection methods may also alter detectability (Jeppsson *et al*. 2010). Thus, non‐resident species and those subject to taxonomic revision since 1970 were excluded from the analysis. We also excluded species for which recording methodologies changed (e.g. most Sesiidae were excluded because the recent introduction of pheromone lures has greatly improved detection rates) and species that occurred in <10 grid squares in the 1970–1999 period, as no range margin could be determined for these species (see next section). This left 673 species (10 462 519 records in total) for our analysis.

Each species occurrence was attributed to a 10 × 10 km grid square of the GB Ordnance Survey (OS) National Grid (hereafter ‘grid squares’) for analysis. The records cover 93% of GB grid squares.

### Classification of southern, northern and widespread species

**Figure 1:**
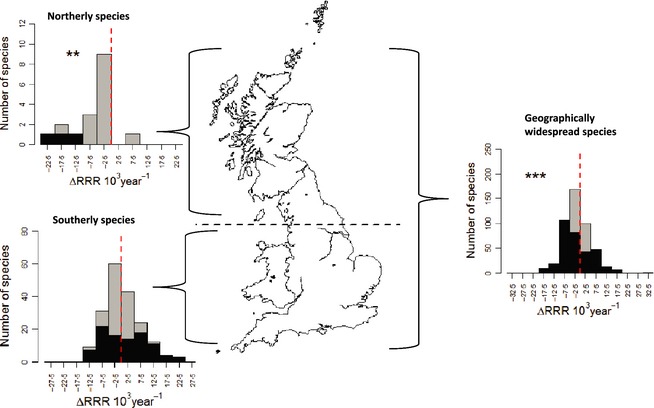
Change in frequency of occurrence (per year change in relative reporting rate, RRR) 1970–1999 vs. 2000–2010 for southerly distributed, northerly distributed and geographically widespread moths. Significant results shown as ** *P *<* *0·01 and *** *P *<* *0·001. Species with individually significant changes (*P *<* *0·05) are shown in black. Change values are multiplied by 10^3^ to improve axis legibility.

Range margins were determined as the mean latitude of the 10 most northerly or southerly occupied grid squares in 1970–1999 (Hickling *et al*. 2006), the baseline period for our analysis. Species were then classified into three groups, based on the 488 km north gridline (OS National Grid). ‘Southern species’ had a northern (cold) range margin that occurred in the southern half of Britain (i.e. south of 488 km north OS). ‘Northern species’ had a southern (warm) range margin north of 488 km north. ‘Widespread species’ did not meet either criteria, occurring in both northern and southern GB (Fig. 1). There was little evidence of taxonomic bias between these groups (Fig. S1, Supporting information).

### Analysis of changes in frequency of occurrence

Temporal and spatial variation in recording intensity (Boakes *et al*. 2010) must be accounted for in analyses of species occurrence data (Ponder *et al*. 2001; Hedenäs *et al*. 2002; Telfer, Preston & Rothery 2002; Hassall & Thompson 2010; Pardo *et al*. 2013). We interpreted moth occurrence data using the program Frescalo to determine temporal trends for each species (Hill 2012). This method utilizes the presence or absence of ‘benchmark’ species to assess recording intensity at a given location. A local set of benchmark species was defined for each (focal) grid square, based on species occurrence data in surrounding ‘neighbourhoods’. The fraction of benchmark species observed in a focal square enables recording effort to be estimated, which can then be used to adjust the observed frequencies of species occurrence. The adjusted frequencies are then used to assess trends over time (see Hill 2012 and Appendix S1, Supporting information for detailed explanation).

Frescalo was applied to the total moth data set (673 species), split into two time periods of roughly equal numbers of records, 1970–1999 vs. 2000–2010. For each time period, a grid square was categorized as having species detected (1) or not‐detected (0) (giving a sample of 720 969 data points). Neighbourhoods were defined based on spatial proximity and floristic similarity using 1970 onwards vascular plant data from Preston, Pearman & Dines (2002). For each location in our analysis, the corresponding neighbourhood was defined as the 50 most floristically similar (using a spatial smoothing kernel) grid squares selected from the 100 geographically closest squares to each location (Appendix S1, Supporting information).

Change in moth species’ frequency of occurrence was estimated by considering the relative reporting rate (RRR; Appendix S1, Supporting information) of each species in each time period (1970–1999 and 2000–2010) (Hill 2012). Temporal trends for each species were expressed as the yearly change in RRR, calculated as the overall change between the mid‐points of the two time periods (i.e. 1984 and 2005, respectively) divided by the number of intervening years. The significance of these trends was determined using a *z*‐test by:

$$z\;=\;\frac{t_{2}-t_{1}}{\sqrt{\sigma_{2}^{2}\;+\;\sigma_{1}^{2}}}$$

where *t*_1_ and *t*_2_ are the relative reporting rates of a given species from the first and second time periods, and ${\sigma_{1}}^{2}$ and ${\sigma_{2}}^{2}$ are the variances associated with the RRR for periods *t*_1_ and *t*_2,_ respectively. Trends in RRR were determined to be significant (at the 95% confidence level) if |*z*| > 1·96. The analyses of Frescalo trends were carried out in R v2.9.2 (R Development Core Team 2009).

Finally, for widespread species, RRR trends were recalculated separately for the northern and southern halves of Britain, dividing the data along the 488 km north gridline.

### Correlation with host plant and environmental variables

We tested host plant effects for the subset of 56 GB macro‐moths that are monophagous (Skinner 2009; Waring, Townsend & Lewington 2009) on vascular plant species for which distribution and trait (Ellenberg indicator values) data were available. Long‐term GB distribution changes in the plants (1930–1960 vs. 1987–1999) and Ellenberg values were derived from PLANTATT (Hill, Preston & Roy 2004). We used all Ellenberg values in PLANTATT (soil nitrogen, soil pH, soil moisture and shade tolerance) excluding salt tolerance, for which there was insufficient variation for the plants in our analysis.

We tested whether changes in frequency of occurrence (ΔRRR year^−1^) of the 56 moth species were correlated with distribution change in their host plants. We fitted a multiple regression of moth changes against their host's Ellenberg values for light, moisture, reaction (pH) and nitrogen. In all these statistical models, we included species distribution grouping (‘southern’ or ‘widespread’ species; no northern species were part of the monophagous group) as a control variable. Regressions were fitted in R with moth ΔRRR year^−1^ as a response variable and either plant distribution change or Ellenberg traits as explanatory variables. Initially, model residuals did not conform to normality, so three outlying data points were removed to rectify this (Shapiro test for normality of residuals: *W* = 0·9776, *P *=* *0·42, *n* = 53), although results were qualitatively similar when including these data. We considered the phylogenetic non‐independence of species by fitting a mixed‐effects model with genus and family as random effects. Higher‐level phylogenetic relationships are not well resolved in Lepidoptera so a full comparative analysis using a phylogeny was not possible (Mutanen, Wahlberg & Kaila 2010). We used the *lme4* and *lmerTest* packages (Bates, Maechler & Dai 2008; with significance of variables assessed using Satterthwaite's approximation for degrees of freedom, Kuznetsova, Brockhoff & Christensen 2013).

## Results

British macro‐moth species decreased significantly in frequency of occurrence between the periods 1970–1999 and 2000–2010 (Wilcoxon signed‐rank test on ΔRRR year^−1^ using all species: *V* = 87 558, *n* = 673, *P *<* *0·001): 260 of the 673 species exhibited significant declines (*P *<* *0·05), with a further 157 species showing a tendency to decline. In contrast, 160 species increased significantly (*P *<* *0·05) in frequency of occurrence, with 96 others showing a tendency to increase. Thus, 420 (62%) of the species have undertaken significant changes in frequency, with 1·6 times as many decreasing as increasing (Table S1, Supporting information). The magnitude of these changes was relatively similar between groups (median ΔRRR year^−1^ for significantly increasing species = 0·006 [range 0·002–0·033]; significantly declining species: median = −0·006 [range = −0·024 to −0·002]; Table S1, Supporting information). The results reveal a wide diversity of occurrence changes among moths.

Geographically limited species showed contrasting trends (Fig. 1). Species restricted to northern Britain (trailing edges of distributions) declined significantly in frequency of occurrence (with 94% of species declining; *V* = 10, *n* = 17, *P *=* *0·002). In contrast, species confined to southern GB did not show a significant change overall (*V* = 8575, *n* = 186, *P *=* *0·87): 24% of species declined significantly, while 27% increased significantly.

**Figure 2:**
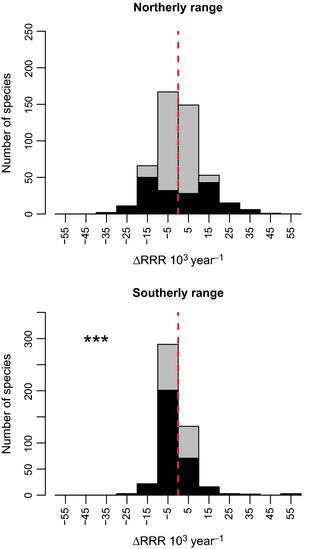
Change in the frequency of occurrence (per year change in relative reporting rate, RRR) 1970–1999 vs. 2000–2010 of geographically widespread moth species in the northern and southern halves of Britain (divided by 488 km north OS gridline, see Fig. 1). Significant result shown as *** *P *≤* *0·001. Species with individually significant changes (*P *<* *0·05) are shown in black. Change values are multiplied by 10^3^ to improve axis legibility.

On average, geographically widespread species decreased in frequency of occurrence (*V* = 39 066, *n* = 470, *P *<* *0·001; Fig. 1): 45% of individual species in this group declined significantly. When trends for widespread species were recalculated separately for southern and northern GB, we found disproportionately larger declines in the south (Fig. 2). There was no significant change in frequency of occurrence of widespread species in northern GB (*V* = 53 569, *n* = 470, *P *=* *0·55), but a significant decline in the south (*V* = 37 017, *n* = 470, *P *≤ 0·001).

**Figure 3:**
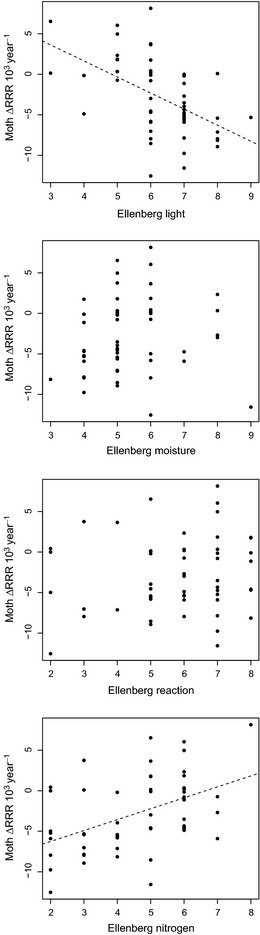
Change in the frequency of occurrence (per year change in relative reporting rate, RRR) 1970–1999 vs. 2000–2010 of monophagous moth species in relation to host plant Ellenberg indicator values. Change values are multiplied by 10^3^ to improve axis legibility. Dashed lines are from univariate regressions.

Changes in frequency of occurrence of monophagous macro‐moths and distribution changes in their larval host plants were not significantly linked (linear regression: slope = 0·002, *t* = 1·33, *P *=* *0·19, *R* ^2^ = 0·03; mixed model: slope = 0·002, *t* = 1·99, *P *=* *0·057; *n* = 53 species for both; Fig. S2, Supporting information). However, there was a negative relationship between moth species’ trends and their host plant Ellenberg light values and a positive correlation between moth trends and host Ellenberg nitrogen values (Table 1; Fig. 3). Moths utilizing larval host plants growing in open, low‐fertility conditions declined over time compared to species using plants in more shaded, nitrogen‐rich environments. There were no relationships between moth trends and Ellenberg values for moisture or reaction.

**Table 1:** Relationships from a multiple regression and linear mixed model of host plant Ellenberg indicator values on change in frequency of occurrence of monophagous moth species (*n* = 53 for both). Significant results (*P *<* *0·05) shown in bold text. Species distribution grouping (‘southern’ or ‘ubiquitous’ species; no northern species were part of the 53 species) was included as a covariate, with the intercept representing southern species

Coefficient	Model 1 multiple regression				Model 2 mixed effects (phylogenetic control)			
	Coefficient	SE	*t*	*P*	Coefficient	SE	*t*	*P*
Intercept	0·0057	0·0050	1·14	0·261	0·0042	0·0049	0·849	0·401
Light	−0·0014	0·0005	−2·64	**0·011**	−0·0011	0·0005	−2·179	**0·035**
Moisture	−0·0007	0·0006	−1·20	0·236	−0·0006	0·0005	−1·139	0·261
Reaction	−0·0004	0·0005	−0·89	0·378	−0·0007	0·0005	−1·432	0·160
Nitrogen	0·0013	0·0006	2·32	**0·025**	0·0015	0·0005	2·772	**0·008**
Species distribution grouping	0·0006	0·0012	0·52	0·607	0·0006	0·0012	0·477	0·636

## Discussion

Macro‐moth species in Great Britain decreased overall in frequency of occurrence between 1970–1999 and 2000–2010, in keeping with a significant decrease in GB macro‐moth abundance over a similar period (Conrad *et al*. 2006), moth distribution trends in other countries (Mattila *et al*. 2008; Groenendijk & Ellis 2011), and declines in other insect taxa (Warren *et al*. 2001; Cameron *et al*. 2011). It provides further evidence that invertebrates are as negatively impacted by environmental change as vertebrates (Thomas *et al*. 2004; Collen *et al*. 2012). The diversity of trends suggests that combinations of different drivers are resulting in a mixture of responses.

The occurrence trends were calculated using the Frescalo method to control for spatiotemporal variation in recorder effort (Hill 2012). Without controlling for this bias, variation in the intensity of recording can confound assessments of species occurrence over time. The method estimated frequency of occurrence, which is a function of both local abundance and distribution extent (Appendix S1, Figs S3 and S4, Supporting information).

The Frescalo method makes a number of assumptions. One is that the probability of finding a species in a locality can be estimated by its frequency in the neighbourhood (floristically similar grid squares in close spatial proximity). We believe this is reasonable because moth species tend to be associated with specific ecotypes and plant communities and because plant communities are generally good indicators of a range of local environmental conditions (e.g. soil structure, pH, moisture levels and microclimate; Ellenberg 1979). A second potential consideration of the Frescalo method is that poorly recorded neighbourhoods cannot provide information about local species frequency. This was not an issue in the current analysis of moth data at 10‐km resolution with neighbourhoods of 50 grid squares, but it could be if analyses were conducted at finer spatiotemporal scales. Finally, the Frescalo method may have limited applicability for less speciose taxonomic groups that have few potential benchmark species.

Our results demonstrate different patterns of change in the frequency of occurrence among macro‐moths with different geographical distributions and host plant traits, providing full or partial support for each of our hypotheses. Moths as a whole decreased in frequency of occurrence, as did northern and geographically widespread species, while southerly distributed species showed no overall trend. Additional analyses showed that geographically widespread species only decreased in the southern half of Britain and showed no overall trend in the north. Correlations between trends of monophagous moths and Ellenberg indicator values of their host plants revealed mixed findings.

The development of an understanding of the drivers of moth biodiversity change in GB is a vital step for conservation biologists and practitioners. We propose an interpretation of our findings based on two major drivers of change for GB biodiversity: habitat modification and climate change. There is growing indirect evidence of the impacts of these drivers on GB moths (Merckx *et al*. 2012; Fox 2013), but we acknowledge that other factors may be involved and drive changes in the occurrence of individual species.

The overall decrease in moth frequencies, and that of the subset of geographically widespread species, is consistent with a response to high levels of habitat modification, as for butterflies (Warren *et al*. 2001), although it does not exclude other explanations.

Our second set of hypotheses related to the performances of three geographically defined groups of moths. Southerly distributed (warmth associated) species were predicted to increase in response to regional climate warming (Fig. S5, Supporting information), but they also inhabit the parts of GB with the highest levels of land‐use change. Some of these species increased and others decreased (resulting in no overall significant trend in this group, Fig. 1). This might reflect a diversity of habitat and climatic sensitivities, although such results could also be due to the species being insensitive to recent changes in climate and land use.

In northern Britain, cold‐adapted species have declined, a response consistent with synergistic negative effects of climate change and habitat modification (as found for four northern GB butterfly species, Franco *et al*. 2006). This is in keeping with other studies implicating climate change in the retraction of warm range margins of cold‐adapted Lepidoptera (Thomas, Franco & Hill 2006; Chen *et al*. 2011; Dieker, Drees & Assmann 2011). Specific conservation measures may be required for these trailing edge populations (Hampe & Petit 2005), including steps to minimize negative land‐use impacts and the protection of climatic refugia.

Geographically widespread species only decreased, on average, in southern GB; population monitoring has yielded similar findings (Conrad *et al*. 2006; Fox *et al*. 2011). Almost all of the widespread species also occur in warmer parts of Europe and are unlikely therefore to have experienced a climatic deterioration of conditions in southern GB, although there may be exceptions (e.g. *Arctia caja* Conrad, Woiwod & Perry 2002) due, for example, to local climatic adaptation. A greater proportion of widespread species is increasing in northern GB (Fig. 2) perhaps reflecting the positive impacts of climate change for some species.

Southern GB has undergone greater loss of semi‐natural habitats since the early 20th century than the north. Comparison of 10‐km grid square resolution land cover data for 1931–1941 with 2000 data suggests an increase in arable and urban land of 20% and 6%, respectively, in southern GB, and a 4% decrease in arable and 1% increase in urban land in the north (T. Jucker pers. comm.; Jucker 2010). Although these habitat conversion trends have slowed recently, the overall pattern of greater habitat modification in the south has been retained and ongoing degradation in habitat quality (e.g. loss of botanical species richness in linear features) has been recorded (Haines‐Young *et al*. 2003; Carey *et al*. 2008). We suggest that the decline of widespread moth species in southern GB is predominantly linked to habitat modification. Further research is needed to assess whether these rates of decline will cause regional extinctions, and to identify effective conservation strategies in the wider countryside (Kleijn *et al*. 2011).

The variation among species is as revealing as the overall trends (Table S1, Supporting information). Sixteen of the 17 northern species showed a declining trend, suggesting relatively consistent responses to drivers of change. In contrast, many southern species increased significantly while others decreased significantly; a pattern also seen among widespread species. Given that species vary in their habitat associations and likely responsiveness to different elements of climate, it is not surprising that simultaneous habitat and climatic changes generate increases in frequency in some species and declines in others (Menéndez *et al*. 2007).

Much recent research has focussed on species’ traits as predictors of biodiversity decline (Mattila *et al*. 2008; Öckinger *et al*. 2010), but success in explaining climate change responses has been limited (Angert *et al*. 2011). We examined traits of the plant hosts of moths, which are expected to reflect sensitivity to land‐use changes more than the climate (Firbank *et al*. 2008; Kleijn *et al*. 2009).

Surprisingly, we found no significant relationship between changes in host plant distributions and frequency of occurrence of dependent moths (Fig. S2, Supporting information). However, specialist moths rarely occupy the entire range of their larval hosts (Quinn, Gaston & Roy 1997), and change in host plant distribution might occur in parts of the range unoccupied by the associated moth. In addition, thresholds of host plant abundance, quality and local distribution may determine moth persistence (Menéndez & Thomas 2000), but these are not accounted for in assessments of distribution change. Finally, the lack of association may stem from the inherent differences in the measures being compared (frequency of occurrence change for moths vs. distribution change for plants).

We did find significant correlations between changes in the frequency of occurrence of moth species and Ellenberg values of host plants for two predictors, showing that monophagous moths that utilize plant species associated with high light intensity and low‐fertility soils tended to decrease most strongly (as have plants with these traits, Carey *et al*. 2008). Decreases among plants and their specialist herbivores associated with open, nutrient‐poor conditions can be attributed to habitat modification directly, through changing agricultural and woodland management, and also indirectly, for example due to eutrophication of the environment (Warren & Key 1991; Firbank *et al*. 2008; Kleijn *et al*. 2009; Payne *et al*. 2013). Such impacts, mediated through botanical communities (Payne *et al*. 2013), have rarely been recorded among herbivores (Hendriks *et al*. 2013). Although enrichment may be reversible on individual sites, new approaches to the management of nutrients in the wider countryside will be required to address declines of species restricted to low‐nutrient environments (Robertson & Vitousek 2009).

Synergistic climate change interactions, both negative and positive, may also occur. Warmer conditions extend the growing season (Menzel & Fabrian 1999) leading to increased plant growth, particularly if coupled with rising soil fertility. Thus, climate change could favour shade‐tolerant species and could, perversely, reduce warm microclimatic niches required by invertebrates (WallisDeVries & van Swaay 2006; Oliver *et al*. 2012). On the other hand, for moth species that utilize plants favoured in high‐nitrogen environments, eutrophication may facilitate climate‐driven range expansion (Betzholtz *et al*. 2012).

Understanding species’ responses to the drivers of biodiversity change is vital to develop adaptive conservation strategies (Mawdsley, O'Malley & Ojima 2009). The diverse patterns of change revealed by our study suggest that drivers of trends are likely to differ between species, necessitating multifaceted approaches to conservation. Nevertheless, a generic solution is to maintain existing high‐quality habitats and create new areas (Lawton *et al*. 2010). This will minimize declines (e.g. of widespread species in the south) and maximize increases (e.g. of southern species), regardless of whether species are responding most strongly, or in combination, to land‐use or climatic changes. Hence, conservation strategies should aim to retain sufficient quantity and quality of habitat to minimize negative synergistic effects (Oliver *et al*. 2010; Araújo *et al*. 2011), while facilitating the exploitation of opportunities created by climate warming (Hodgson *et al*. 2011; Thomas *et al*. 2012). This requires the protection of remaining habitats from deleterious impacts, but also sufficient knowledge of land management techniques to maximize habitat quality. Such knowledge is limited for moths but can start by identifying landscape elements and management practices associated with enhanced species richness and abundance (Fuentes‐Montemayor, Goulson & Park 2011; Merckx *et al*. 2012).

## Supporting information


**Appendix S1.** Further information on the Frescalo methodology used to assess moth trends.

**Fig. S1.** The frequency and proportion of moths in different distribution groupings by taxonomic family.

**Fig. S2.** Change in frequency of occurrence of monophagous moth species in relation to change in host plant distribution.

**Fig. S3.** Relationship between number of occupied grid squares and relative reporting rate.

**Fig. S4.** Relationship between change in frequency of occurrence and proportional change in occupied grid squares.

**Fig. S5.** Annual accumulated temperatures (growing degree days >5 °C) during the two recording periods.

**Table S1.** Species frequency of occurrence trends with confidence limits.
