# Supplementary material for: Long‐term changes to the frequency of occurrence of British moths are consistent with opposing and synergistic effects of climate and land‐use changes
Source: J Appl Ecol. 2014 Apr 29;51(4):949–57. doi: 10.1111/1365-2664.12256 (PMC4413814; doi:10.1111/1365-2664.12256)
Supplement: Supplementary file 1 — Appendix S1. Further information on the Frescalo methodology used to assess moth trends. [file JPE-51-949-s001.doc]

**Appendix S1.**  **Frescalo analysis of distribution change**

Frescalo is a method that can be applied to species occurrence data to assess variation in recorder effort and produce trends in species frequency over time (Hill 2012). The method requires two parameters to be identified by users, although sensitivity analyses suggest that precise values are not critical (see supporting Information in Hill 2012).The first parameter is the standard neighbourhood frequency Φ, which generally reflects how well the species group is recorded. The default value of Φ is 0.74, but the Frescalo program provides an output value of phi using a convergence algorithm which scales local neighbourhood frequencies by sampling effort multipliers. For groups that are not completely recorded, Hill (2012) suggests setting Φ greater than the 98^th^ percentile of observed values of local neighbourhood frequency. Therefore, for the analysis of the entire Great Britain (GB) macro-moth dataset we increased the value of Φ to 0.89, to remain above this 98^th^ percentile. For the analysis where northern and southern halves of geographically widespread species distributions were assessed separately we used a Φ value of 0.94 for southern hectads (10km x 10km grid squares), which were relatively better recorded than northern hectads, where we set the value of Φ to 0.74.

The second input parameter required by Frescalo is the R*, the proportion of species treated as benchmark species. The most common species in a local neighbourhood are used as ‘benchmarks’ to give an indication of how well recorded a given hectad is, and this is then used to modify the reporting frequency of the focal species in order to inform on the probability of occurrence. Ideally, the benchmark species should be relatively stable in frequency over time, but sensitivity analyses have shown that the method is robust to dynamic benchmark species (see supporting Information in Hill 2012). We retained the default value of 0.27 for this parameter.

Following the methods in Hill (2012), the probability of reporting species in a given hectad in a given time period depends on 1) recorder effort, which is measured as the proportion of benchmark species reported from the hectad in that time period, 2) the time-independent probability of the species occurring in that hectad and 3) the Relative Reporting Rate (RRR) for the time period. The RRR is the ratio of the rate at which the focal species is reported to the rate at which the benchmark species are reported within the range of occupancy of the focal species in a given time period. In order that values of RRR for rare species are of comparable magnitude to those for commoner species, this is relativized by dividing by the time-independent probability of finding the focal species, also within its range of occupancy. Relative Reporting Rate is obtained iteratively through the formula given in Hill (2012). Hence, although it is a measure without units, the change in Relative Reporting Rate (ΔRRR) between time periods describes temporal changes in the estimated species frequency across all hectads, i.e. a negative ΔRRR indicates a decline in species frequency.

As described in the main paper, the significance of these trends can be determined by:


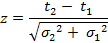


where *t_1_* and *t_2_* are the Relative Reporting Rates of a given species from the first and second time periods and *σ_1_^2^* and *σ_2_* ^2^ are the variances associated with the RRR for periods *t_1_* and *t_2_* respectively.

The Frescalo method estimates species’ frequency of occurrence, which is a function of both local abundance and distribution extent. In practice, these two variables are inextricably linked because the probability of a species being recorded in a grid square depends both on local abundance and on recording effort. In theory, changes in frequency of occurrence could relate solely to variation in local abundance with no distribution change. However, because we aggregated data over broad time periods in our study (thereby increasing recording effort), and the fact that changes in frequency of occurrence calculated for macro-moths correlated significantly with the raw number of occupied grid squares (Figs S3 & S4), it is likely that changes in frequency described here also reflect changes in moth distribution extents in GB.

**References**

Hill, M.O. (2012) Local frequency as a key to interpreting species occurrence data when recording effort is not known. *Methods in Ecology and Evolution*, **3**, 195–205.
