# Supplementary material for: Long‐term changes to the frequency of occurrence of British moths are consistent with opposing and synergistic effects of climate and land‐use changes
Source: J Appl Ecol. 2014 Apr 29;51(4):949–57. doi: 10.1111/1365-2664.12256 (PMC4413814; doi:10.1111/1365-2664.12256)

**Figure S1.** The frequency, panel (a), and proportion, panel (b), of moths in different distribution groupings (northerly, southerly, geographically widespread) by taxonomic family.


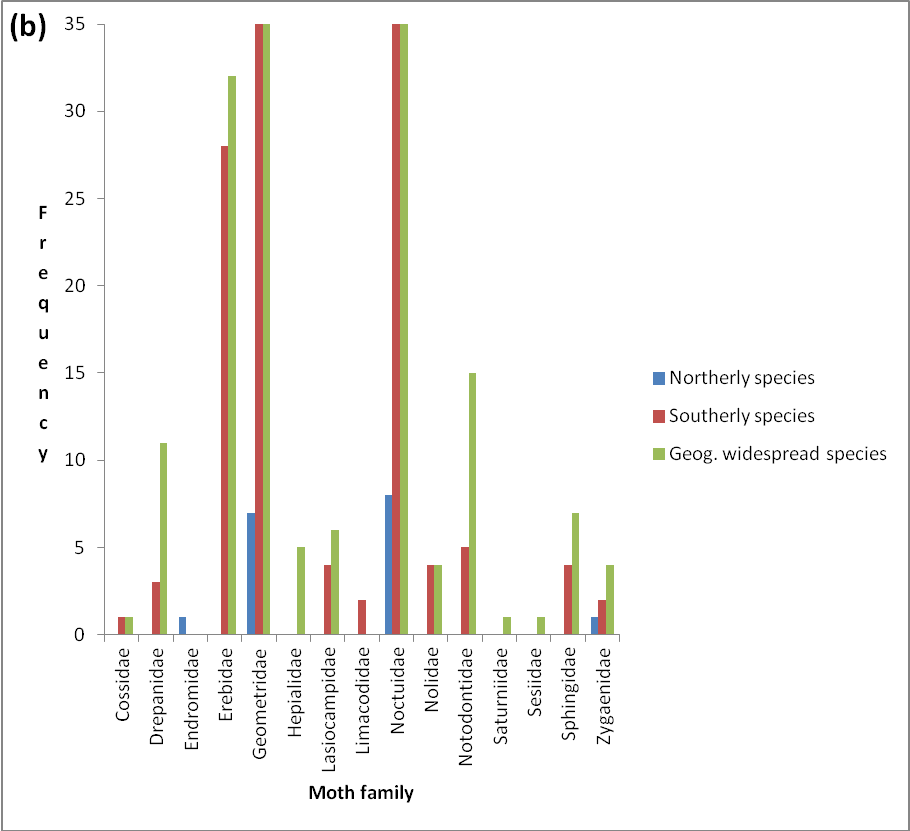

Supplement: Supplementary file 2 — Fig. S1. The frequency and proportion of moths in different distribution groupings by taxonomic family. [file JPE-51-949-s002.doc]
