# Supplementary material for: Long‐term changes to the frequency of occurrence of British moths are consistent with opposing and synergistic effects of climate and land‐use changes
Source: J Appl Ecol. 2014 Apr 29;51(4):949–57. doi: 10.1111/1365-2664.12256 (PMC4413814; doi:10.1111/1365-2664.12256)

**Figure S2.** Change in the frequency of occurrence of monophagous moth species 1970-99 versus 2000-10 in relation to change in host plant distribution between 1970 and 1987 (with outliers removed as per main text).


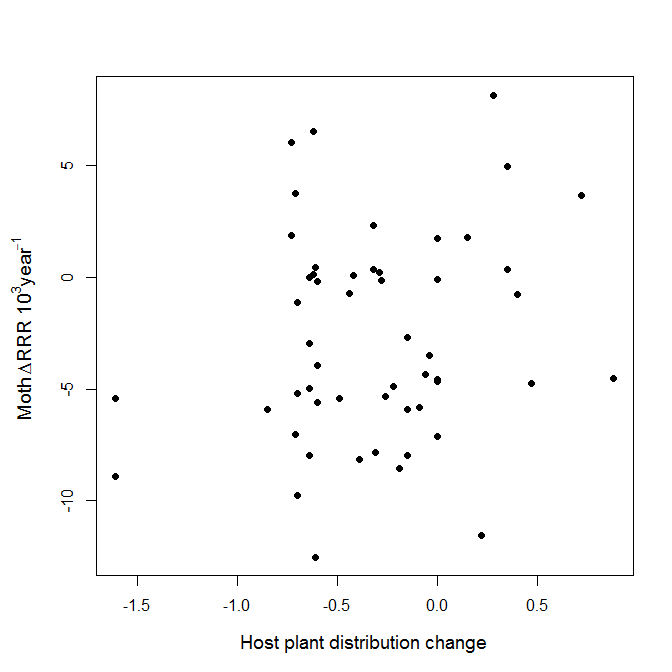

Supplement: Supplementary file 3 — Fig. S2. Change in frequency of occurrence of monophagous moth species in relation to change in host plant distribution. [file JPE-51-949-s003.doc]
