# Supplementary material for: Long‐term changes to the frequency of occurrence of British moths are consistent with opposing and synergistic effects of climate and land‐use changes
Source: J Appl Ecol. 2014 Apr 29;51(4):949–57. doi: 10.1111/1365-2664.12256 (PMC4413814; doi:10.1111/1365-2664.12256)

**Figure S3.** Relationship between number of hectads (grid squares) occupied by each species in (a) 1970-99 and (b) 2000-10 versus the relative reporting rate (RRR) which indicates frequency of occurrence after standardising for recorder effort. The curved relationships show that the most common species is approximately 2.5 times more common than the average benchmark species, whilst rare species are only approximately 0.25 times as common as the average benchmark species.


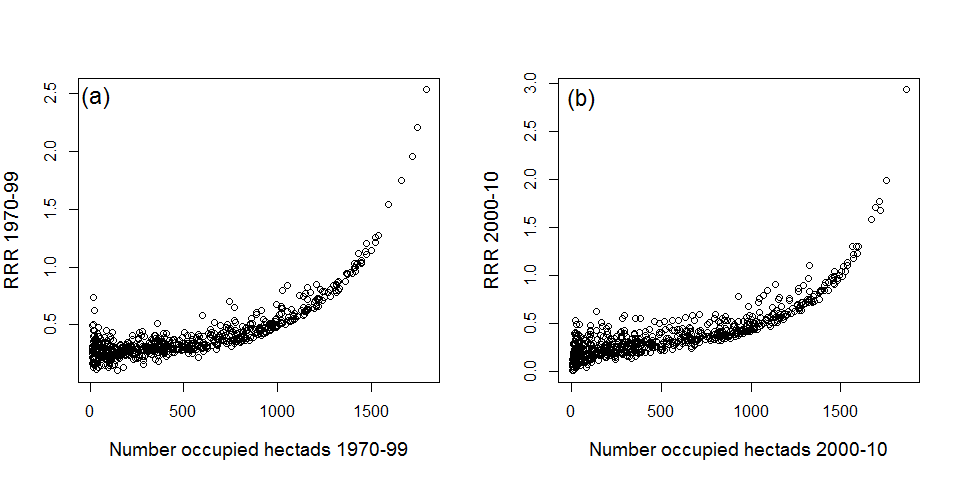

Supplement: Supplementary file 4 — Fig. S3. Relationship between number of occupied grid squares and relative reporting rate. [file JPE-51-949-s004.doc]
