# Supplementary material for: Long‐term changes to the frequency of occurrence of British moths are consistent with opposing and synergistic effects of climate and land‐use changes
Source: J Appl Ecol. 2014 Apr 29;51(4):949–57. doi: 10.1111/1365-2664.12256 (PMC4413814; doi:10.1111/1365-2664.12256)

**Figure S4.** Panel (a) shows the relationship between change in moth frequency of occurrence (ΔRRR) using the Frescalo method which controls for spatiotemporal variation in recorder effort versus proportional change in hectads (grid squares) listed as occupied. The time periods under consideration are 1970-99 versus 2000-2010. The correlation is significant (F_1,161_ = 1301, p < 0.001). We select two outliers from this relationship, both with large positive RRR trends, to demonstrate how the Frescalo method accounts for variation in recorder effort (shown in panel (b)). Panel (a) suggests that the species *T. cupressata* has a greater increase in records than expected from changes in frequency relative to benchmark species (which inform on recorder effort). Hence, the large increase in number of records is partly due to increased recording effort in these locations in the latter period. In contrast, *R. sericealis* has fewer records than expected which indicates relatively less recording effort focussed in areas where this species occurs. Panel (b) shows the recording intensity in hectads newly occupied by the two moths in the latter period. Recording intensity is calculated as the proportion of benchmarks species recorded in a hectad. Hence, this confirms that *T. cupressata* has expanded into well recorded areas, whilst *R. sericealis* has expanded into poorly recorded areas.


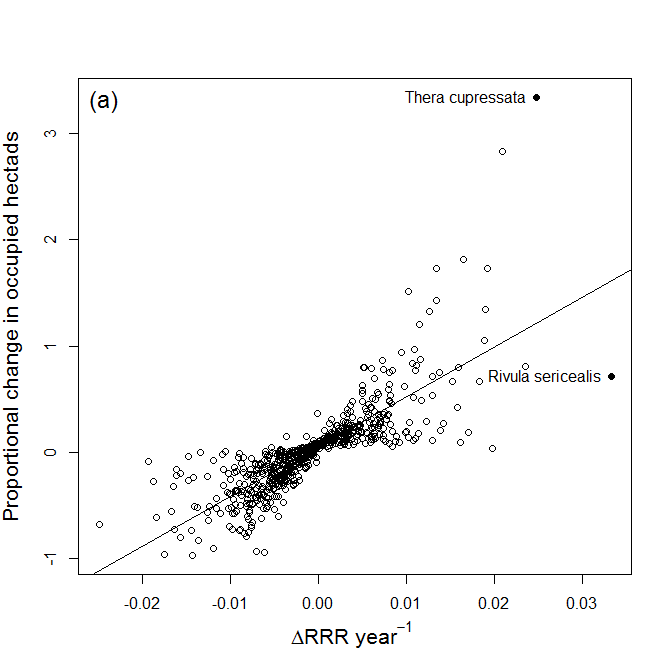


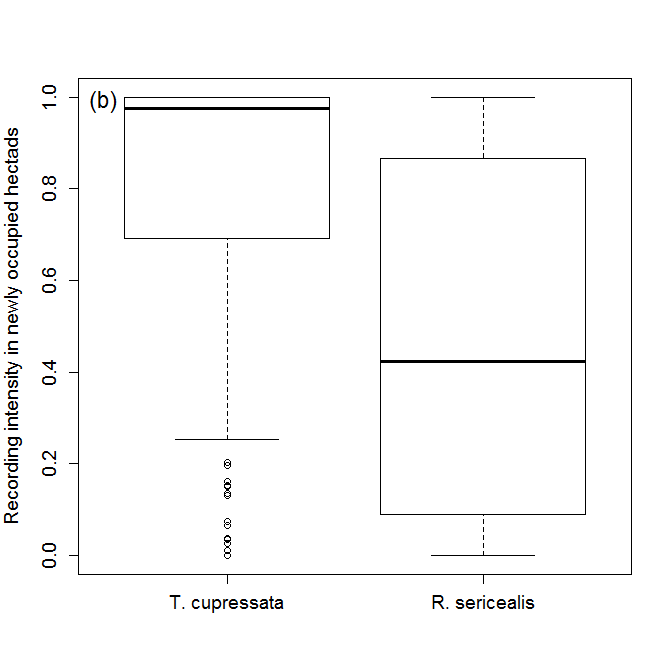

Supplement: Supplementary file 5 — Fig. S4. Relationship between change in frequency of occurrence and proportional change in occupied grid squares. [file JPE-51-949-s005.doc]
