# Supplementary material for: Long‐term changes to the frequency of occurrence of British moths are consistent with opposing and synergistic effects of climate and land‐use changes
Source: J Appl Ecol. 2014 Apr 29;51(4):949–57. doi: 10.1111/1365-2664.12256 (PMC4413814; doi:10.1111/1365-2664.12256)

**Figure S5.** Annual accumulated temperatures (growing degree days > 5°C; GDD5; calculated from daily mean temperature data from the UK Met Office Central England Temperature dataset; <http://www.metoffice.gov.uk/hadobs/hadcet/>) during the two recording periods. The box and whisker plots show median annual GDD5, upper and lower quartiles and 95^th^ percentiles. Annual GDD5 values were significantly higher in the latter period (t-test: t = 2.84, df = 17.71, p = 0.01).


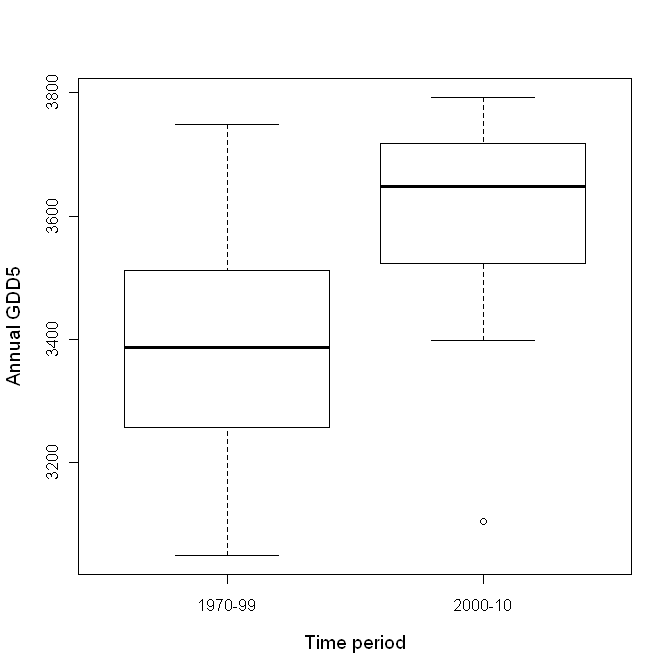

Supplement: Supplementary file 6 — Fig. S5. Annual accumulated temperatures (growing degree days >5 °C) during the two recording periods. [file JPE-51-949-s006.doc]
