# Supplementary material for: Long‐term changes to the frequency of occurrence of British moths are consistent with opposing and synergistic effects of climate and land‐use changes
Source: J Appl Ecol. 2014 Apr 29;51(4):949–57. doi: 10.1111/1365-2664.12256 (PMC4413814; doi:10.1111/1365-2664.12256)
Supplement: Supplementary file 7 — Table S1. Species frequency of occurrence trends with confidence limits. [file JPE-51-949-s007.pdf]

Table S1. Individual species results for 673 resident* GB macro-moths 1970-99 versus 2000-10 showing changes in grid square (hectad) occupancy and frequency of occurrence (RRR) with confidence limits.

Taxon	Vernacular	Hectads occupied in 1970-99	Hectads occupied in 2000-10	Proportional change occupied hectads	RRR 1970-99	St. Dev	RRR 2000-10	St. Dev	Δ RRR/year	z	Significance
Abraxas grossulariata	Magpie Moth	1220	1164	-0.046	0.732	0.026	0.558	0.019	-0.0085	-5.40	p<0.05
Abraxas sylvata	Clouded Magpie	366	286	-0.219	0.289	0.016	0.207	0.013	-0.0040	-3.98	p<0.05
Abrostola tripartita	Spectacle	1206	1457	0.208	0.666	0.023	0.951	0.035	0.0139	6.81	p<0.05
Abrostola triplasia	Dark Spectacle	535	561	0.049	0.292	0.013	0.288	0.013	-0.0002	-0.22	NS
Acasis viretata	Yellow-barred Brindle	621	907	0.461	0.302	0.013	0.472	0.018	0.0083	7.66	p<0.05
Achlya flavicornis	Yellow Horned	535	589	0.101	0.283	0.013	0.298	0.013	0.0007	0.82	NS
Acronicta aceris	Sycamore	451	593	0.315	0.371	0.019	0.53	0.026	0.0078	4.94	p<0.05
Acronicta alni	Alder Moth	573	585	0.021	0.326	0.014	0.295	0.013	-0.0015	-1.62	NS
Acronicta euphorbiae	Sweet Gale Moth	22	19	-0.136	0.169	0.038	0.142	0.033	-0.0013	-0.54	NS
Acronicta leporina	Miller	730	845	0.158	0.343	0.014	0.388	0.015	0.0022	2.19	p<0.05
Acronicta megacephala	Poplar Grey	818	955	0.167	0.446	0.017	0.52	0.02	0.0036	2.82	p<0.05
Acronicta menyanthidis	Light Knot Grass	186	121	-0.349	0.271	0.02	0.158	0.015	-0.0055	-4.52	p<0.05
Acronicta psi	Grey Dagger	1029	913	-0.113	0.516	0.018	0.37	0.013	-0.0071	-6.58	p<0.05
Acronicta rumicis	Knot Grass	1057	1166	0.103	0.536	0.019	0.568	0.02	0.0016	1.16	NS
Acronicta tridens	Dark Dagger	391	297	-0.240	0.313	0.016	0.208	0.012	-0.0051	-5.25	p<0.05
Actebia praecox	Portland Moth	61	18	-0.705	0.218	0.029	0.062	0.015	-0.0076	-4.78	p<0.05
Adscita geryon	Cistus Forester	72	52	-0.278	0.307	0.038	0.2	0.029	-0.0052	-2.24	p<0.05
Adscita statices	Forester	146	100	-0.315	0.225	0.019	0.14	0.014	-0.0041	-3.60	p<0.05
Aethalura punctulata	Grey Birch	544	450	-0.173	0.34	0.015	0.249	0.012	-0.0044	-4.74	p<0.05
Agriopis aurantiaria	Scarce Umber	653	495	-0.242	0.326	0.013	0.215	0.01	-0.0054	-6.77	p<0.05
Agriopis leucophaearia	Spring Usher	489	472	-0.035	0.283	0.013	0.26	0.012	-0.0011	-1.30	NS
Agriopis marginaria	Dotted Border	963	928	-0.036	0.455	0.016	0.377	0.013	-0.0038	-3.78	p<0.05
Agrochola circellaris	Brick	828	854	0.031	0.368	0.014	0.348	0.013	-0.0010	-1.05	NS
Agrochola helvola	Flounced Chestnut	491	319	-0.350	0.291	0.014	0.16	0.009	-0.0064	-7.87	p<0.05
Agrochola litura	Brown-spot Pinion	777	659	-0.152	0.448	0.018	0.322	0.013	-0.0061	-5.67	p<0.05
Agrochola lota	Red-line Quaker	874	1010	0.156	0.388	0.014	0.437	0.015	0.0024	2.39	p<0.05
Agrochola lychnidis	Beaded Chestnut	813	794	-0.023	0.49	0.02	0.413	0.016	-0.0038	-3.01	p<0.05
Agrochola macilenta	Yellow-line Quaker	804	970	0.206	0.346	0.013	0.414	0.015	0.0033	3.43	p<0.05
Agrotis cinerea	Light Feathered Rustic	139	68	-0.511	0.297	0.026	0.129	0.016	-0.0082	-5.50	p<0.05
Agrotis clavis	Heart and Club	586	667	0.138	0.355	0.016	0.388	0.016	0.0016	1.46	NS
Agrotis exclamationis	Heart and Dart	1476	1450	-0.018	1.203	0.047	0.98	0.037	-0.0109	-3.73	p<0.05
Agrotis puta	Shuttle-shaped Dart	888	1066	0.200	0.591	0.024	0.797	0.033	0.0100	5.05	p<0.05
Agrotis ripae	Sand Dart	75	71	-0.053	0.303	0.036	0.268	0.033	-0.0017	-0.72	NS
Agrotis segetum	Turnip Moth	977	979	0.002	0.512	0.019	0.453	0.016	-0.0029	-2.38	p<0.05
Agrotis trux	Crescent Dart	121	112	-0.074	0.437	0.042	0.344	0.034	-0.0045	-1.72	NS
Agrotis vestigialis	Archer's Dart	281	175	-0.377	0.445	0.028	0.233	0.018	-0.0103	-6.37	p<0.05
Alcis jubata	Dotted Carpet	157	162	0.032	0.264	0.022	0.231	0.019	-0.0016	-1.14	NS
Alcis repandata	Mottled Beauty	1409	1464	0.039	1.028	0.039	0.992	0.038	-0.0018	-0.66	NS
Aleucis distinctata	Sloe Carpet	46	26	-0.435	0.481	0.077	0.243	0.05	-0.0116	-2.59	p<0.05
Allophyes oxyacanthae	Green-brindled Crescent	969	990	0.022	0.465	0.017	0.424	0.015	-0.0020	-1.81	NS
Alsophila aescularia	March Moth	929	966	0.040	0.466	0.017	0.438	0.016	-0.0014	-1.20	NS
Amphipoea crinanensis	Crinan Ear	90	67	-0.256	0.231	0.025	0.157	0.019	-0.0036	-2.36	p<0.05
Amphipoea fucosa	Saltern Ear	160	143	-0.106	0.276	0.022	0.238	0.02	-0.0019	-1.28	NS
Amphipoea lucens	Large Ear	227	252	0.110	0.251	0.017	0.257	0.017	0.0003	0.25	NS
Amphipoea oculea	Ear Moth	569	499	-0.123	0.288	0.013	0.23	0.011	-0.0028	-3.41	p<0.05
Amphipyra tragopoginis	Mouse Moth	1220	958	-0.215	0.725	0.026	0.396	0.014	-0.0160	-11.14	p<0.05
Anaplectoides prasina	Green Arches	683	798	0.168	0.346	0.014	0.381	0.014	0.0017	1.77	NS
Anarta cordigera	Small Dark Yellow Underwing	25	8	-0.680	0.628	0.147	0.118	0.043	-0.0249	-3.33	p<0.05
Anarta melanopa	Broad-bordered White Underwing	20	21	0.050	0.231	0.056	0.224	0.057	-0.0003	-0.09	NS
Anarta myrtilli	Beautiful Yellow Underwing	329	227	-0.310	0.285	0.016	0.179	0.012	-0.0052	-5.30	p<0.05
Angerona prunaria	Orange Moth	176	150	-0.148	0.304	0.024	0.218	0.018	-0.0042	-2.87	p<0.05
Anticlea badiata	Shoulder Stripe	881	885	0.005	0.43	0.016	0.388	0.014	-0.0020	-1.98	p<0.05
Anticlea derivata	Streamer	790	932	0.180	0.364	0.014	0.423	0.015	0.0029	2.88	p<0.05

Anticollix sparsata	Dentated Pug	42	20	-0.524	0.404	0.064	0.167	0.038	-0.0116	-3.18	p<0.05
Antitype chi	Grey Chi	476	312	-0.345	0.365	0.018	0.202	0.012	-0.0080	-7.53	p<0.05
Apamea anceps	Large Nutmeg	369	360	-0.024	0.338	0.019	0.298	0.017	-0.0020	-1.57	NS
Apamea crenata	Clouded-bordered Brindle	1200	1279	0.066	0.642	0.022	0.641	0.022	0.0000	-0.03	NS
Apamea epomidion	Clouded Brindle	525	537	0.023	0.301	0.014	0.285	0.013	-0.0008	-0.84	NS
Apamea furva	Confused	213	109	-0.488	0.281	0.02	0.129	0.013	-0.0074	-6.37	p<0.05
Apamea lithoxylaea	Light Arches	1158	1154	-0.003	0.637	0.023	0.551	0.019	-0.0042	-2.88	p<0.05
Apamea monoglypha	Dark Arches	1746	1755	0.005	2.202	0.096	1.987	0.091	-0.0105	-1.63	NS
Apamea oblonga	Crescent Striped	124	53	-0.573	0.365	0.034	0.137	0.019	-0.0111	-5.85	p<0.05
Apamea ophiogramma	Double Lobed	544	572	0.051	0.314	0.014	0.307	0.013	-0.0003	-0.37	NS
Apamea remissa	Dusky Brocade	1139	1147	0.007	0.579	0.02	0.513	0.017	-0.0032	-2.51	p<0.05
Apamea scolopacina	Slender Brindle	605	722	0.193	0.328	0.014	0.391	0.016	0.0031	2.96	p<0.05
Apamea sordens	Rustic Shoulder-knot	963	858	-0.109	0.516	0.019	0.384	0.014	-0.0064	-5.59	p<0.05
Apamea sublustris	Reddish Light Arches	186	183	-0.016	0.291	0.022	0.274	0.021	-0.0008	-0.56	NS
Apamea unanimis	Small Clouded Brindle	549	615	0.120	0.291	0.013	0.31	0.013	0.0009	1.03	NS
Apamea zeta	Exile	48	47	-0.021	0.249	0.037	0.222	0.033	-0.0013	-0.54	NS
Apeira syringaria	Lilac Beauty	606	587	-0.031	0.345	0.015	0.3	0.013	-0.0022	-2.27	p<0.05
Aplocera efformata	Lesser Treble-bar	323	291	-0.099	0.329	0.019	0.277	0.017	-0.0025	-2.04	p<0.05
Aplocera plagiata	Treble-bar	822	685	-0.167	0.404	0.015	0.28	0.011	-0.0060	-6.67	p<0.05
Apocheima hispidaria	Small Brindled Beauty	254	217	-0.146	0.285	0.019	0.242	0.017	-0.0021	-1.69	NS
Apoda limacodes	Festoon	86	139	0.616	0.232	0.026	0.432	0.04	0.0098	4.19	p<0.05
Aporophyla australis	Feathered Brindle	41	47	0.146	0.336	0.055	0.308	0.047	-0.0014	-0.39	NS
Aporophyla nigra	Black Rustic	773	1046	0.353	0.342	0.013	0.505	0.018	0.0080	7.34	p<0.05
Archanara algae	Rush Wainscot	18	15	-0.167	0.292	0.07	0.223	0.059	-0.0034	-0.75	NS
Archanara dissoluta	Brown-veined Wainscot	206	210	0.019	0.317	0.023	0.308	0.022	-0.0004	-0.28	NS
Archanara geminipuncta	Twin-spotted Wainscot	218	279	0.280	0.287	0.02	0.351	0.022	0.0031	2.15	p<0.05
Archanara sparganii	Webb's Wainscot	116	206	0.776	0.244	0.023	0.47	0.035	0.0110	5.40	p<0.05
Archiearis notha	Light Orange Underwing	56	33	-0.411	0.227	0.031	0.134	0.024	-0.0045	-2.37	p<0.05
Archiearis parthenias	Orange Underwing	340	331	-0.026	0.271	0.015	0.259	0.015	-0.0006	-0.57	NS
Arctia caja	Garden Tiger	1311	1107	-0.156	0.795	0.028	0.465	0.016	-0.0161	-10.23	p<0.05
Arctia villica	Cream-spot Tiger	195	183	-0.062	0.332	0.025	0.248	0.019	-0.0041	-2.68	p<0.05
Arenostola phragmitidis	Fen Wainscot	260	275	0.058	0.372	0.025	0.366	0.024	-0.0003	-0.17	NS
Aspitates gilvaria	Straw Belle	13	10	-0.231	0.25	0.071	0.171	0.055	-0.0039	-0.88	NS
Asteroscopus sphinx	Sprawler	383	356	-0.070	0.29	0.016	0.248	0.014	-0.0020	-1.98	p<0.05
Asthena albulata	Small White Wave	439	450	0.025	0.301	0.015	0.283	0.014	-0.0009	-0.88	NS
Atethmia centrago	Centre-barred Sallow	822	1016	0.236	0.381	0.014	0.505	0.018	0.0060	5.44	p<0.05
Atolmis rubricollis	Red-necked Footman	179	489	1.732	0.134	0.01	0.409	0.02	0.0134	12.30	p<0.05
Autographa bractea	Gold Spangle	504	404	-0.198	0.438	0.021	0.308	0.016	-0.0063	-4.92	p<0.05
Autographa jota	Plain Golden Y	1129	1012	-0.104	0.622	0.022	0.444	0.016	-0.0087	-6.54	p<0.05
Autographa pulchrina	Beautiful Golden Y	1330	1271	-0.044	0.817	0.029	0.632	0.021	-0.0090	-5.17	p<0.05
Axylia putris	Flame	1149	1290	0.123	0.775	0.03	0.899	0.035	0.0060	2.69	p<0.05
Bena bicolorana	Scarce Silver-lines	468	587	0.254	0.291	0.014	0.363	0.016	0.0035	3.39	p<0.05
Biston betularia	Peppered Moth	1324	1432	0.082	0.874	0.032	0.932	0.035	0.0028	1.22	NS
Biston strataria	Oak Beauty	715	814	0.138	0.368	0.015	0.405	0.016	0.0018	1.69	NS
Blepharita adusta	Dark Brocade	560	385	-0.313	0.337	0.015	0.197	0.01	-0.0068	-7.77	p<0.05
Brachionycha nubeculosa	Rannoch Sprawler	10	18	0.800	0.221	0.074	0.329	0.086	0.0053	0.95	NS
Brachylomia viminalis	Minor Shoulder-knot	802	561	-0.300	0.402	0.015	0.23	0.01	-0.0084	-9.54	p<0.05
Bupalus piniaria	Bordered White	900	785	-0.128	0.465	0.017	0.349	0.013	-0.0057	-5.42	p<0.05
Cabera exanthemata	Common Wave	1188	1305	0.098	0.65	0.023	0.707	0.025	0.0028	1.68	NS
Cabera pusaria	Common White Wave	1375	1408	0.024	0.934	0.034	0.847	0.031	-0.0042	-1.89	NS
Callimorpha dominula	Scarlet Tiger	212	271	0.278	0.338	0.025	0.405	0.027	0.0033	1.82	NS
Callistege mi	Mother Shipton	666	633	-0.050	0.347	0.014	0.298	0.012	-0.0024	-2.66	p<0.05
Calliteara pudibunda	Pale Tussock	862	1093	0.268	0.557	0.022	0.849	0.036	0.0142	6.92	p<0.05
Calophasia lunula	Toadflax Brocade	16	45	1.813	0.156	0.04	0.494	0.078	0.0165	3.86	p<0.05
Campaea margaritata	Light Emerald	1376	1513	0.100	0.943	0.035	1.099	0.043	0.0076	2.81	p<0.05
Camptogramma bilineata	Yellow Shell	1504	1379	-0.083	1.145	0.043	0.75	0.026	-0.0193	-7.86	p<0.05
Caradrina morpheus	Mottled Rustic	1051	1028	-0.022	0.613	0.023	0.519	0.019	-0.0046	-3.15	p<0.05
Carsia sororiata	Manchester Treble-bar	103	75	-0.272	0.249	0.025	0.177	0.021	-0.0035	-2.21	p<0.05
Catarhoe cuculata	Royal Mantle	137	108	-0.212	0.299	0.026	0.198	0.02	-0.0049	-3.08	p<0.05

Catarhoe rubidata	Ruddy Carpet	157	142	-0.096	0.257	0.021	0.198	0.017	-0.0029	-2.18	p<0.05
Catocala fraxini	Clifden Nonpareil	40	39	-0.025	0.165	0.027	0.155	0.025	-0.0005	-0.27	NS
Catocala nupta	Red Underwing	666	717	0.077	0.427	0.018	0.43	0.018	0.0001	0.12	NS
Catocala promissa	Light Crimson Underwing	13	9	-0.308	0.218	0.062	0.16	0.054	-0.0028	-0.71	NS
Celaena haworthii	Haworth's Minor	281	220	-0.217	0.317	0.02	0.223	0.015	-0.0046	-3.76	p<0.05
Celaena leucostigma	Crescent	476	461	-0.032	0.286	0.014	0.25	0.012	-0.0018	-1.95	NS
Cepphis advenaria	Little Thorn	90	75	-0.167	0.369	0.041	0.293	0.035	-0.0037	-1.41	NS
Cerapteryx graminis	Antler Moth	1252	1007	-0.196	0.788	0.027	0.467	0.016	-0.0157	-10.23	p<0.05
Cerastis leucographa	White-marked	123	107	-0.130	0.272	0.025	0.219	0.022	-0.0026	-1.59	NS
Cerastis rubricosa	Red Chestnut	928	958	0.032	0.423	0.015	0.393	0.014	-0.0015	-1.46	NS
Cerura vinula	Puss Moth	857	668	-0.221	0.393	0.015	0.243	0.01	-0.0073	-8.32	p<0.05
Charanyca trigrammica	Treble Lines	688	865	0.257	0.391	0.016	0.531	0.021	0.0068	5.30	p<0.05
Charissa obscurata	Annulet	219	109	-0.502	0.32	0.022	0.139	0.013	-0.0088	-7.08	p<0.05
Chesias legatella	Streak	493	363	-0.264	0.315	0.015	0.216	0.012	-0.0048	-5.15	p<0.05
Chesias rufata	Broom-tip	169	97	-0.426	0.296	0.023	0.15	0.015	-0.0071	-5.32	p<0.05
Chiasmia clathrata	Latticed Heath	927	761	-0.179	0.551	0.021	0.361	0.014	-0.0093	-7.53	p<0.05
Chilodes maritimus	Silky Wainscot	198	224	0.131	0.293	0.021	0.312	0.022	0.0009	0.62	NS
Chlorissa viridata	Small Grass Emerald	48	25	-0.479	0.277	0.041	0.124	0.025	-0.0075	-3.19	p<0.05
Chloroclysta citrata	Dark Marbled Carpet	990	799	-0.193	0.544	0.019	0.35	0.013	-0.0095	-8.43	p<0.05
Chloroclysta concinnata	Arran Carpet	15	3	-0.800	0.398	0.112	0.078	0.046	-0.0156	-2.64	p<0.05
Chloroclysta miata	Autumn Green Carpet	397	305	-0.232	0.286	0.015	0.195	0.011	-0.0044	-4.89	p<0.05
Chloroclysta siterata	Red-green Carpet	578	1185	1.050	0.22	0.009	0.607	0.021	0.0189	16.94	p<0.05
Chloroclysta truncata	Common Marbled Carpet	1413	1571	0.112	0.998	0.037	1.219	0.049	0.0108	3.60	p<0.05
Chloroclystis v-ata	V-Pug	857	1000	0.167	0.471	0.018	0.539	0.02	0.0033	2.53	p<0.05
Chortodes brevilinea	Fenn's Wainscot	12	9	-0.250	0.498	0.151	0.294	0.102	-0.0100	-1.12	NS
Chortodes elymi	Lyme Grass	39	24	-0.385	0.428	0.073	0.222	0.047	-0.0100	-2.37	p<0.05
Chortodes extrema	Concolorous	18	22	0.222	0.319	0.079	0.375	0.084	0.0027	0.49	NS
Chortodes fluxa	Mere Wainscot	106	91	-0.142	0.404	0.041	0.307	0.033	-0.0047	-1.84	NS
Chortodes pygmina	Small Wainscot	836	945	0.130	0.382	0.014	0.414	0.015	0.0016	1.56	NS
Cidaria fulvata	Barred Yellow	1086	1058	-0.026	0.569	0.02	0.478	0.017	-0.0044	-3.47	p<0.05
Cilix glaucata	Chinese Character	1020	1048	0.027	0.659	0.025	0.618	0.023	-0.0020	-1.21	NS
Cleora cinctaria	Ringed Carpet	35	27	-0.229	0.313	0.055	0.234	0.046	-0.0039	-1.10	NS
Cleorodes lichenaria	Brussels Lace	237	332	0.401	0.264	0.018	0.332	0.019	0.0033	2.60	p<0.05
Clostera curtula	Chocolate-tip	410	524	0.278	0.319	0.017	0.438	0.021	0.0058	4.40	p<0.05
Clostera pigra	Small Chocolate-tip	114	39	-0.658	0.229	0.022	0.076	0.012	-0.0075	-6.11	p<0.05
Coenobia rufa	Small Rufous	288	450	0.563	0.231	0.014	0.37	0.018	0.0068	6.10	p<0.05
Coenocalpe lapidata	Slender-striped Rufous	19	18	-0.053	0.222	0.052	0.193	0.05	-0.0014	-0.40	NS
Colocasia coryli	Nut-tree Tussock	678	888	0.310	0.377	0.016	0.516	0.02	0.0068	5.43	p<0.05
Colostygia multistrigaria	Mottled Grey	497	499	0.004	0.289	0.013	0.263	0.012	-0.0013	-1.47	NS
Colostygia olivata	Beech-green Carpet	171	94	-0.450	0.27	0.021	0.129	0.014	-0.0069	-5.59	p<0.05
Colostygia pectinataria	Green Carpet	1430	1589	0.111	1.032	0.039	1.298	0.053	0.0130	4.04	p<0.05
Colotois pennaria	Feathered Thorn	932	1003	0.076	0.44	0.016	0.439	0.015	0.0000	-0.05	NS
Comibaena bajularia	Blotched Emerald	372	331	-0.110	0.357	0.02	0.292	0.017	-0.0032	-2.48	p<0.05
Conistra ligula	Dark Chestnut	552	659	0.194	0.289	0.013	0.342	0.014	0.0026	2.77	p<0.05
Conistra rubiginea	Dotted Chestnut	101	199	0.970	0.195	0.02	0.418	0.032	0.0109	5.91	p<0.05
Conistra vaccinii	Chestnut	941	1160	0.233	0.434	0.016	0.566	0.02	0.0064	5.15	p<0.05
Coscinia cribraria	Speckled Footman	13	4	-0.692	0.294	0.084	0.088	0.045	-0.0100	-2.16	p<0.05
Cosmia affinis	Lesser-spotted Pinion	270	204	-0.244	0.277	0.018	0.188	0.013	-0.0043	-4.01	p<0.05
Cosmia diffinis	White-spotted Pinion	102	24	-0.765	0.218	0.022	0.047	0.01	-0.0083	-7.08	p<0.05
Cosmia pyralina	Lunar-spotted Pinion	397	339	-0.146	0.39	0.021	0.286	0.016	-0.0051	-3.94	p<0.05
Cosmia trapezina	Dun-bar	1215	1282	0.055	0.726	0.026	0.721	0.026	-0.0002	-0.14	NS
Cosmorhoe ocellata	Purple Bar	1218	1190	-0.023	0.697	0.024	0.575	0.02	-0.0060	-3.91	p<0.05
Cossus cossus	Goat Moth	104	74	-0.288	0.152	0.015	0.098	0.012	-0.0026	-2.81	p<0.05
Craniophora ligustri	Coronet	362	658	0.818	0.233	0.013	0.462	0.02	0.0112	9.60	p<0.05
Crocallis elinguaria	Scalloped Oak	1322	1348	0.020	0.863	0.032	0.777	0.028	-0.0042	-2.02	p<0.05
Cryphia domestica	Marbled Beauty	933	998	0.070	0.504	0.019	0.512	0.019	0.0004	0.30	NS
Cryphia muralis	Marbled Green	200	277	0.385	0.346	0.026	0.421	0.028	0.0037	1.96	p<0.05
Cucullia absinthii	Wormwood	119	67	-0.437	0.287	0.027	0.153	0.019	-0.0065	-4.06	p<0.05
Cucullia asteris	Star-wort	109	65	-0.404	0.372	0.038	0.194	0.025	-0.0087	-3.91	p<0.05

Cucullia chamomillae	Chamomile Shark	397	283	-0.287	0.303	0.016	0.189	0.011	-0.0056	-5.87	p<0.05
Cucullia umbratica	Shark	798	686	-0.140	0.387	0.015	0.276	0.011	-0.0054	-5.97	p<0.05
Cybosia mesomella	Four-dotted Footman	374	371	-0.008	0.327	0.018	0.299	0.016	-0.0014	-1.16	NS
Cyclophora albipunctata	Birch Mocha	256	251	-0.020	0.283	0.018	0.255	0.017	-0.0014	-1.13	NS
Cyclophora annularia	Mocha	111	164	0.477	0.211	0.021	0.313	0.026	0.0050	3.05	p<0.05
Cyclophora linearia	Clay Triple-lines	326	487	0.494	0.248	0.014	0.382	0.018	0.0065	5.88	p<0.05
Cyclophora pendularia	Dingy Mocha	21	19	-0.095	0.162	0.036	0.157	0.037	-0.0002	-0.10	NS
Cyclophora porata	False Mocha	98	31	-0.684	0.219	0.022	0.063	0.011	-0.0076	-6.34	p<0.05
Cyclophora punctaria	Maiden's Blush	397	589	0.484	0.274	0.014	0.441	0.02	0.0081	6.84	p<0.05
Cymatophorima diluta	Oak Lutestring	222	164	-0.261	0.277	0.019	0.199	0.016	-0.0038	-3.14	p<0.05
Dasypolia templi	Brindled Ochre	187	138	-0.262	0.268	0.02	0.18	0.016	-0.0043	-3.44	p<0.05
Deilephila elpenor	Elephant Hawk-moth	1197	1412	0.180	0.694	0.025	0.927	0.035	0.0114	5.42	p<0.05
Deilephila porcellus	Small Elephant Hawk-moth	621	823	0.325	0.305	0.013	0.42	0.016	0.0056	5.58	p<0.05
Deileptenia ribeata	Satin Beauty	262	387	0.477	0.218	0.014	0.298	0.016	0.0039	3.76	p<0.05
Deltote bankiana	Silver Barred	17	24	0.412	0.29	0.073	0.401	0.086	0.0054	0.98	NS
Deltote uncula	Silver Hook	190	129	-0.321	0.325	0.024	0.195	0.018	-0.0063	-4.33	p<0.05
Diachrysia chrysitis	Burnished Brass	1450	1482	0.022	1.054	0.039	0.965	0.036	-0.0043	-1.68	NS
Diachrysia chryson	Scarce Burnished Brass	32	28	-0.125	0.262	0.048	0.207	0.041	-0.0027	-0.87	NS
Diacrisia sannio	Clouded Buff	247	233	-0.057	0.288	0.019	0.252	0.017	-0.0018	-1.41	NS
Diaphora mendica	Muslin Moth	795	857	0.078	0.439	0.017	0.451	0.017	0.0006	0.50	NS
Diarsia brunnea	Purple Clay	961	912	-0.051	0.476	0.017	0.387	0.014	-0.0043	-4.04	p<0.05
Diarsia dahlii	Barred Chestnut	305	196	-0.357	0.29	0.017	0.169	0.012	-0.0059	-5.81	p<0.05
Diarsia mendica	Ingrailed Clay	1447	1429	-0.012	1.028	0.038	0.845	0.03	-0.0089	-3.78	p<0.05
Diarsia rubi	Small Square-spot	1324	1481	0.119	0.797	0.028	0.949	0.035	0.0074	3.39	p<0.05
Dicallomera fascelina	Dark Tussock	134	122	-0.090	0.25	0.022	0.224	0.021	-0.0013	-0.85	NS
Dichonia aprilina	Merveille du Jour	603	766	0.270	0.273	0.012	0.344	0.013	0.0035	4.01	p<0.05
Dicycla oo	Heart Moth	17	9	-0.471	0.177	0.044	0.096	0.032	-0.0040	-1.49	NS
Diloba caeruleocephala	Figure of Eight	646	311	-0.519	0.436	0.019	0.155	0.009	-0.0137	-13.37	p<0.05
Discestra trifolii	Nutmeg	747	663	-0.112	0.454	0.018	0.337	0.014	-0.0057	-5.13	p<0.05
Discoloxia blomeri	Blomer's Rivulet	112	119	0.063	0.248	0.024	0.245	0.023	-0.0001	-0.09	NS
Drepana falcataria	Pebble Hook-tip	954	1084	0.136	0.476	0.017	0.536	0.019	0.0029	2.35	p<0.05
Drymonia dodonaea	Marbled Brown	419	451	0.076	0.331	0.017	0.322	0.016	-0.0004	-0.39	NS
Drymonia ruficornis	Lunar Marbled Brown	610	758	0.243	0.32	0.014	0.405	0.016	0.0041	4.00	p<0.05
Dryobotodes eremita	Brindled Green	605	683	0.129	0.306	0.013	0.333	0.014	0.0013	1.41	NS
Dypterygia scabriuscula	Bird's Wing	365	353	-0.033	0.368	0.021	0.344	0.02	-0.0012	-0.83	NS
Dyscia fagaria	Grey Scalloped Bar	151	72	-0.523	0.238	0.02	0.11	0.013	-0.0062	-5.37	p<0.05
Earias clorana	Cream-bordered Green Pea	184	293	0.592	0.266	0.02	0.429	0.027	0.0080	4.85	p<0.05
Ecliptopera silaceata	Small Phoenix	1167	1352	0.159	0.649	0.023	0.801	0.029	0.0074	4.11	p<0.05
Ectropis bistortata	Engrailed	994	1178	0.185	0.491	0.018	0.591	0.021	0.0049	3.62	p<0.05
Ectropis crepuscularia	Small Engrailed	518	243	-0.531	0.34	0.016	0.13	0.008	-0.0102	-11.74	p<0.05
Egira conspicillaris	Silver Cloud	42	38	-0.095	0.344	0.058	0.299	0.051	-0.0022	-0.58	NS
Eilema caniola	Hoary Footman	54	105	0.944	0.229	0.032	0.423	0.044	0.0095	3.57	p<0.05
Eilema complana	Scarce Footman	634	803	0.267	0.43	0.019	0.599	0.025	0.0082	5.38	p<0.05
Eilema depressa	Buff Footman	364	854	1.346	0.185	0.01	0.574	0.023	0.0190	15.51	p<0.05
Eilema griseola	Dingy Footman	515	931	0.808	0.299	0.014	0.781	0.034	0.0235	13.11	p<0.05
Eilema lurideola	Common Footman	1117	1323	0.184	0.758	0.029	1.108	0.047	0.0171	6.34	p<0.05
Eilema pygmaeola	Pigmy Footman	19	27	0.421	0.36	0.087	0.485	0.1	0.0061	0.94	NS
Eilema sororcula	Orange Footman	146	560	2.836	0.104	0.009	0.532	0.025	0.0209	16.11	p<0.05
Elaphria venustula	Rosy Marbled	97	106	0.093	0.337	0.036	0.386	0.039	0.0024	0.92	NS
Electrophaes corylata	Broken-barred Carpet	916	893	-0.025	0.464	0.017	0.394	0.014	-0.0034	-3.18	p<0.05
Ematurga atomaria	Common Heath	835	702	-0.159	0.478	0.018	0.335	0.013	-0.0070	-6.44	p<0.05
Enargia paleacea	Angle-striped Sallow	135	140	0.037	0.266	0.024	0.246	0.021	-0.0010	-0.63	NS
Endromis versicolora	Kentish Glory	23	14	-0.391	0.303	0.067	0.17	0.047	-0.0065	-1.63	NS
Ennomos alniaria	Canary-shouldered Thorn	1086	1184	0.090	0.581	0.021	0.609	0.021	0.0014	0.94	NS
Ennomos autumnaria	Large Thorn	106	104	-0.019	0.316	0.032	0.283	0.029	-0.0016	-0.76	NS
Ennomos erosaria	September Thorn	640	411	-0.358	0.391	0.017	0.2	0.01	-0.0093	-9.68	p<0.05
Ennomos fuscantaria	Dusky Thorn	704	829	0.178	0.403	0.017	0.474	0.019	0.0035	2.78	p<0.05
Ennomos quercinaria	August Thorn	509	465	-0.086	0.324	0.015	0.256	0.012	-0.0033	-3.54	p<0.05
Entephria caesiata	Grey Mountain Carpet	360	160	-0.556	0.513	0.029	0.171	0.014	-0.0167	-10.62	p<0.05

Entephria flavicinctata	Yellow-ringed Carpet	29	29	0.000	0.25	0.048	0.228	0.045	-0.0011	-0.33	NS
Epione repandaria	Bordered Beauty	590	618	0.047	0.312	0.014	0.296	0.013	-0.0008	-0.84	NS
Epirrhoe alternata	Common Carpet	1662	1717	0.033	1.75	0.073	1.775	0.079	0.0012	0.23	NS
Epirrhoe galiata	Galium Carpet	323	263	-0.186	0.307	0.018	0.213	0.013	-0.0046	-4.23	p<0.05
Epirrhoe rivata	Wood Carpet	300	230	-0.233	0.279	0.017	0.181	0.012	-0.0048	-4.71	p<0.05
Epirrhoe tristata	Small Argent & Sable	270	162	-0.400	0.339	0.022	0.176	0.014	-0.0080	-6.25	p<0.05
Epirrita autumnata	Autumnal Moth	436	356	-0.183	0.301	0.015	0.226	0.012	-0.0037	-3.90	p<0.05
Epirrita christyi	Pale November Moth	309	230	-0.256	0.284	0.016	0.195	0.013	-0.0043	-4.32	p<0.05
Epirrita dilutata	November Moth	796	666	-0.163	0.377	0.014	0.265	0.011	-0.0055	-6.29	p<0.05
Epirrita filigrammaria	Small Autumnal Moth	225	116	-0.484	0.302	0.021	0.143	0.013	-0.0078	-6.44	p<0.05
Erannis defoliaria	Mottled Umber	955	832	-0.129	0.453	0.016	0.328	0.012	-0.0061	-6.25	p<0.05
Eremobia ochroleuca	Dusky Sallow	599	633	0.057	0.579	0.028	0.559	0.026	-0.0010	-0.52	NS
Eriogaster lanestris	Small Eggar	103	88	-0.146	0.167	0.017	0.137	0.015	-0.0015	-1.32	NS
Euchoeca nebulata	Dingy Shell	398	478	0.201	0.256	0.013	0.304	0.014	0.0023	2.51	p<0.05
Euclidia glyphica	Burnet Companion	492	474	-0.037	0.343	0.017	0.315	0.015	-0.0014	-1.24	NS
Eugnorisma depuncta	Plain Clay	104	80	-0.231	0.263	0.026	0.177	0.02	-0.0042	-2.62	p<0.05
Eugnorisma glareosa	Autumnal Rustic	797	838	0.051	0.431	0.016	0.419	0.016	-0.0006	-0.53	NS
Eulithis mellinata	Spinach	690	436	-0.368	0.394	0.016	0.201	0.01	-0.0094	-10.23	p<0.05
Eulithis populata	Northern Spinach	771	599	-0.223	0.654	0.027	0.397	0.017	-0.0125	-8.05	p<0.05
Eulithis prunata	Phoenix	749	837	0.117	0.369	0.014	0.397	0.015	0.0014	1.36	NS
Eulithis pyraliata	Barred Straw	1314	1315	0.001	0.837	0.03	0.73	0.026	-0.0052	-2.70	p<0.05
Eulithis testata	Chevron	841	695	-0.174	0.454	0.017	0.31	0.012	-0.0070	-6.92	p<0.05
Euphyia biangulata	Cloaked Carpet	154	166	0.078	0.279	0.023	0.281	0.023	0.0001	0.06	NS
Euphyia unangulata	Sharp-angled Carpet	328	292	-0.110	0.341	0.02	0.259	0.016	-0.0040	-3.20	p<0.05
Eupithecia abbreviata	Brindled Pug	772	1044	0.352	0.343	0.013	0.504	0.018	0.0079	7.25	p<0.05
Eupithecia abietaria	Cloaked Pug	46	32	-0.304	0.217	0.032	0.159	0.028	-0.0028	-1.36	NS
Eupithecia absinthiata	Wormwood Pug	788	931	0.181	0.357	0.014	0.412	0.015	0.0027	2.68	p<0.05
Eupithecia assimilata	Currant Pug	526	697	0.325	0.252	0.011	0.332	0.013	0.0039	4.70	p<0.05
Eupithecia centaureata	Lime-speck Pug	856	929	0.085	0.51	0.02	0.526	0.02	0.0008	0.57	NS
Eupithecia distinctaria	Thyme Pug	63	23	-0.635	0.255	0.033	0.092	0.019	-0.0080	-4.28	p<0.05
Eupithecia dodoneata	Oak-tree Pug	442	629	0.423	0.262	0.013	0.384	0.016	0.0060	5.92	p<0.05
Eupithecia egenaria	Pauper Pug	14	25	0.786	0.217	0.059	0.34	0.069	0.0060	1.35	NS
Eupithecia exiguata	Mottled Pug	679	903	0.330	0.329	0.013	0.471	0.018	0.0069	6.40	p<0.05
Eupithecia expallidata	Bleached Pug	132	80	-0.394	0.288	0.026	0.157	0.018	-0.0064	-4.14	p<0.05
Eupithecia haworthiata	Haworth's Pug	280	292	0.043	0.315	0.02	0.313	0.019	-0.0001	-0.07	NS
Eupithecia icterata	Tawny Speckled Pug	1020	815	-0.201	0.535	0.019	0.327	0.012	-0.0101	-9.26	p<0.05
Eupithecia indigata	Ochreous Pug	264	310	0.174	0.222	0.014	0.255	0.015	0.0016	1.61	NS
Eupithecia innotata	Angle-barred Pug	84	32	-0.619	0.201	0.022	0.07	0.012	-0.0064	-5.23	p<0.05
Eupithecia insigniata	Pinion-spotted Pug	84	47	-0.440	0.336	0.038	0.171	0.025	-0.0080	-3.63	p<0.05
Eupithecia inturbata	Maple Pug	298	321	0.077	0.286	0.017	0.293	0.017	0.0003	0.29	NS
Eupithecia irriguata	Marbled Pug	59	34	-0.424	0.286	0.038	0.141	0.024	-0.0071	-3.23	p<0.05
Eupithecia lariciata	Larch Pug	398	328	-0.176	0.276	0.014	0.209	0.012	-0.0033	-3.63	p<0.05
Eupithecia linariata	Toadflax Pug	501	447	-0.108	0.34	0.016	0.275	0.013	-0.0032	-3.15	p<0.05
Eupithecia millefoliata	Yarrow Pug	78	106	0.359	0.295	0.034	0.413	0.042	0.0058	2.18	p<0.05
Eupithecia nanata	Narrow-winged Pug	795	705	-0.113	0.433	0.016	0.331	0.013	-0.0050	-4.95	p<0.05
Eupithecia phoeniceata	Cypress Pug	119	195	0.639	0.283	0.027	0.45	0.035	0.0081	3.78	p<0.05
Eupithecia pimpinellata	Pimpinel Pug	93	42	-0.548	0.279	0.029	0.118	0.018	-0.0079	-4.72	p<0.05
Eupithecia plumbeolata	Lead-coloured Pug	78	51	-0.346	0.207	0.024	0.131	0.018	-0.0037	-2.53	p<0.05
Eupithecia pulchellata	Foxglove Pug	911	1066	0.170	0.43	0.016	0.505	0.017	0.0037	3.21	p<0.05
Eupithecia pusillata	Juniper Pug	403	319	-0.208	0.312	0.016	0.23	0.013	-0.0040	-3.98	p<0.05
Eupithecia pygmaeata	Marsh Pug	94	69	-0.266	0.199	0.021	0.144	0.018	-0.0027	-1.99	p<0.05
Eupithecia satyrata	Satyr Pug	365	241	-0.340	0.329	0.018	0.2	0.013	-0.0063	-5.81	p<0.05
Eupithecia simpliciata	Plain Pug	299	273	-0.087	0.31	0.019	0.264	0.016	-0.0022	-1.85	NS
Eupithecia subfuscata	Grey Pug	940	986	0.049	0.438	0.016	0.42	0.015	-0.0009	-0.82	NS
Eupithecia subumbrata	Shaded Pug	230	189	-0.178	0.325	0.022	0.25	0.019	-0.0037	-2.58	p<0.05
Eupithecia succenturiata	Bordered Pug	645	513	-0.205	0.46	0.02	0.302	0.014	-0.0077	-6.47	p<0.05
Eupithecia tantillaria	Dwarf Pug	443	439	-0.009	0.284	0.014	0.272	0.013	-0.0006	-0.63	NS
Eupithecia tenuiata	Slender Pug	448	497	0.109	0.264	0.013	0.279	0.013	0.0007	0.82	NS
Eupithecia tripunctaria	White-spotted Pug	672	812	0.208	0.334	0.014	0.395	0.015	0.0030	2.97	p<0.05

Eupithecia trisignaria	Triple-spotted Pug	191	211	0.105	0.234	0.017	0.247	0.017	0.0006	0.54	NS
Eupithecia valerianata	Valerian Pug	113	89	-0.212	0.235	0.022	0.174	0.019	-0.0030	-2.10	p<0.05
Eupithecia venosata	Netted Pug	306	184	-0.399	0.28	0.016	0.153	0.011	-0.0062	-6.54	p<0.05
Eupithecia virgaureata	Golden-rod Pug	229	373	0.629	0.204	0.014	0.308	0.017	0.0051	4.72	p<0.05
Eupithecia vulgata	Common Pug	1108	1241	0.120	0.57	0.02	0.628	0.022	0.0028	1.95	NS
Euplagia quadripunctaria	Jersey Tiger	42	102	1.429	0.2	0.032	0.476	0.053	0.0135	4.46	p<0.05
Euplexia lucipara	Small Angle Shades	1186	1271	0.072	0.651	0.023	0.657	0.023	0.0003	0.18	NS
Euproctis chrysorrhoea	Brown-tail	290	377	0.300	0.31	0.019	0.409	0.023	0.0048	3.32	p<0.05
Euproctis similis	Yellow-tail	1029	1038	0.009	0.8	0.033	0.718	0.029	-0.0040	-1.87	NS
Eupsilia transversa	Satellite	806	948	0.176	0.361	0.014	0.431	0.015	0.0034	3.41	p<0.05
Eurois occulta	Great Brocade	243	328	0.350	0.207	0.014	0.266	0.015	0.0029	2.88	p<0.05
Euthrix potatoria	Drinker	1161	1264	0.089	0.823	0.032	0.862	0.033	0.0019	0.85	NS
Euxoa cursoria	Coast Dart	71	36	-0.493	0.294	0.036	0.158	0.027	-0.0066	-3.02	p<0.05
Euxoa nigricans	Garden Dart	718	355	-0.506	0.408	0.016	0.154	0.008	-0.0124	-14.20	p<0.05
Euxoa obelisca	Square-spot Dart	74	44	-0.405	0.299	0.036	0.171	0.026	-0.0062	-2.88	p<0.05
Euxoa tritici	White-line Dart	582	372	-0.361	0.403	0.018	0.215	0.011	-0.0092	-8.91	p<0.05
Falcaria lacertinaria	Scalloped Hook-tip	659	660	0.002	0.368	0.015	0.34	0.014	-0.0014	-1.36	NS
Furcula bicuspis	Alder Kitten	162	193	0.191	0.244	0.02	0.275	0.02	0.0015	1.10	NS
Furcula bifida	Poplar Kitten	367	384	0.046	0.258	0.014	0.257	0.014	0.0000	-0.05	NS
Furcula furcula	Sallow Kitten	826	959	0.161	0.381	0.014	0.431	0.015	0.0024	2.44	p<0.05
Gastropacha quercifolia	Lappet	331	88	-0.734	0.364	0.021	0.069	0.007	-0.0144	-13.33	p<0.05
Geometra papilionaria	Large Emerald	1019	997	-0.022	0.525	0.019	0.435	0.015	-0.0044	-3.72	p<0.05
Gnophos obfuscata	Scotch Annulet	86	42	-0.512	0.451	0.051	0.163	0.026	-0.0140	-5.03	p<0.05
Gortyna flavago	Frosted Orange	813	979	0.204	0.369	0.014	0.452	0.016	0.0040	3.90	p<0.05
Graphiphora augur	Double Dart	865	332	-0.616	0.51	0.019	0.133	0.007	-0.0184	-18.62	p<0.05
Gymnoscelis rufifasciata	Double-striped Pug	931	1324	0.422	0.423	0.015	0.748	0.026	0.0159	10.83	p<0.05
Habrosyne pyritoides	Buff Arches	1055	1134	0.075	0.844	0.035	0.906	0.039	0.0030	1.18	NS
Hada plebeja	Shears	891	922	0.035	0.419	0.015	0.396	0.014	-0.0011	-1.12	NS
Hadena albimacula	White Spot	26	16	-0.385	0.372	0.075	0.205	0.052	-0.0081	-1.83	NS
Hadena bicruris	Lychnis	994	1047	0.053	0.482	0.017	0.466	0.016	-0.0008	-0.69	NS
Hadena compta	Varied Coronet	327	351	0.073	0.376	0.022	0.385	0.022	0.0004	0.29	NS
Hadena confusa	Marbled Coronet	370	270	-0.270	0.306	0.016	0.193	0.012	-0.0055	-5.65	p<0.05
Hadena luteago	Barrett's Marbled Coronet	19	26	0.368	0.213	0.051	0.211	0.043	-0.0001	-0.03	NS
Hadena perplexa	Tawny Shears	421	326	-0.226	0.313	0.016	0.21	0.012	-0.0050	-5.15	p<0.05
Hadena rivularis	Campion	773	774	0.001	0.363	0.014	0.323	0.012	-0.0020	-2.17	p<0.05
Hecatera bicolorata	Broad-barred White	691	569	-0.177	0.407	0.017	0.275	0.012	-0.0064	-6.34	p<0.05
Heliophobus reticulata	Bordered Gothic	59	4	-0.932	0.154	0.02	0.01	0.005	-0.0070	-6.99	p<0.05
Heliothis maritima	Shoulder-striped Clover	16	7	-0.563	0.452	0.118	0.195	0.075	-0.0125	-1.84	NS
Heliothis viriplaca	Marbled Clover	54	42	-0.222	0.22	0.031	0.155	0.025	-0.0032	-1.63	NS
Hemaris fuciformis	Broad-bordered Bee Hawk	90	74	-0.178	0.201	0.021	0.149	0.018	-0.0025	-1.88	NS
Hemaris tityus	Narrow-bordered Bee Hawk	84	110	0.310	0.148	0.017	0.179	0.018	0.0015	1.25	NS
Hemistola chrysoprasaria	Small Emerald	479	571	0.192	0.363	0.018	0.429	0.02	0.0032	2.45	p<0.05
Hemithea aestivaria	Common Emerald	887	985	0.110	0.611	0.025	0.674	0.027	0.0031	1.71	NS
Hepialus fusconebulosa	Map-winged Swift	732	756	0.033	0.501	0.02	0.485	0.019	-0.0008	-0.58	NS
Hepialus hecta	Gold Swift	707	516	-0.270	0.363	0.014	0.219	0.01	-0.0070	-8.37	p<0.05
Hepialus humuli	Ghost Moth	1211	1180	-0.026	0.663	0.023	0.544	0.019	-0.0058	-3.99	p<0.05
Hepialus lupulinus	Common Swift	1076	1170	0.087	0.633	0.023	0.674	0.025	0.0020	1.21	NS
Hepialus sylvina	Orange Swift	929	1014	0.091	0.476	0.018	0.497	0.018	0.0010	0.82	NS
Herminia grisealis	Small Fan-foot	964	1105	0.146	0.491	0.018	0.562	0.02	0.0035	2.64	p<0.05
Herminia tarsicrinalis	Shaded Fan-foot	12	22	0.833	0.311	0.095	0.531	0.127	0.0107	1.39	NS
Heterogenea asella	Triangle	18	26	0.444	0.261	0.062	0.336	0.067	0.0037	0.82	NS
Hoplodrina alsines	Uncertain	1021	1151	0.127	0.644	0.024	0.746	0.029	0.0050	2.71	p<0.05
Hoplodrina ambigua	Vine's Rustic	453	674	0.488	0.347	0.018	0.588	0.027	0.0118	7.43	p<0.05
Hoplodrina blanda	Rustic	991	1129	0.139	0.531	0.019	0.606	0.022	0.0037	2.58	p<0.05
Horisme tersata	Fern	346	315	-0.090	0.41	0.024	0.314	0.019	-0.0047	-3.14	p<0.05
Horisme vitalbata	Small Waved Umber	363	408	0.124	0.383	0.022	0.419	0.023	0.0018	1.13	NS
Hydraecia micacea	Rosy Rustic	1219	1343	0.102	0.655	0.023	0.715	0.025	0.0029	1.77	NS
Hydraecia petasitis	Butterbur	100	47	-0.530	0.219	0.022	0.098	0.014	-0.0059	-4.64	p<0.05
Hydrelia flammeolaria	Small Yellow Wave	612	754	0.232	0.328	0.014	0.41	0.016	0.0040	3.86	p<0.05

Hydrelia sylvata	Waved Carpet	100	121	0.210	0.211	0.022	0.226	0.021	0.0007	0.49	NS
Hydriomena furcata	July Highflyer	1523	1463	-0.039	1.209	0.046	0.906	0.033	-0.0148	-5.35	p<0.05
Hydriomena impluviata	May Highflyer	680	743	0.093	0.326	0.013	0.333	0.013	0.0003	0.38	NS
Hydriomena ruberata	Ruddy Highflyer	190	100	-0.474	0.238	0.018	0.118	0.012	-0.0059	-5.55	p<0.05
Hylaea fasciaria	Barred Red	892	879	-0.015	0.434	0.016	0.38	0.014	-0.0026	-2.54	p<0.05
Hyles gallii	Bedstraw Hawk-moth	155	128	-0.174	0.24	0.02	0.185	0.017	-0.0027	-2.10	p<0.05
Hyloicus pinastri	Pine Hawk-moth	262	459	0.752	0.238	0.015	0.521	0.028	0.0138	8.91	p<0.05
Hypena crassalis	Beautiful Snout	195	223	0.144	0.309	0.023	0.331	0.023	0.0011	0.68	NS
Hypena proboscidalis	Snout	1472	1596	0.084	1.136	0.043	1.303	0.053	0.0081	2.45	p<0.05
Hypenodes humidalis	Marsh Oblique-barred	101	111	0.099	0.232	0.024	0.243	0.024	0.0005	0.32	NS
Hypomecis punctinalis	Pale Oak Beauty	341	349	0.023	0.406	0.024	0.395	0.023	-0.0005	-0.33	NS
Hypomecis roboraria	Great Oak Beauty	116	111	-0.043	0.285	0.028	0.281	0.028	-0.0002	-0.10	NS
Hyppa rectilinea	Saxon	71	79	0.113	0.259	0.032	0.247	0.029	-0.0006	-0.28	NS
Idaea aversata	Riband Wave	1434	1563	0.090	1.116	0.043	1.306	0.054	0.0093	2.75	p<0.05
Idaea biselata	Small Fan-footed Wave	1223	1410	0.153	0.719	0.026	0.901	0.033	0.0089	4.33	p<0.05
Idaea contiguaria	Weaver's Wave	13	6	-0.538	0.222	0.064	0.121	0.051	-0.0049	-1.23	NS
Idaea dilutaria	Silky Wave	11	3	-0.727	0.276	0.084	0.076	0.047	-0.0098	-2.08	p<0.05
Idaea dimidiata	Single-dotted Wave	1027	1160	0.130	0.643	0.025	0.776	0.03	0.0065	3.41	p<0.05
Idaea emarginata	Small Scallop	410	357	-0.129	0.422	0.023	0.316	0.018	-0.0052	-3.63	p<0.05
Idaea fuscovenosa	Dwarf Cream Wave	446	500	0.121	0.378	0.019	0.408	0.02	0.0015	1.09	NS
Idaea muricata	Purple-bordered Gold	51	33	-0.353	0.304	0.043	0.199	0.035	-0.0051	-1.89	NS
Idaea rusticata	Least Carpet	163	293	0.798	0.261	0.021	0.587	0.039	0.0159	7.36	p<0.05
Idaea seriata	Small Dusty Wave	574	707	0.232	0.323	0.014	0.419	0.017	0.0047	4.36	p<0.05
Idaea straminata	Plain Wave	447	407	-0.089	0.297	0.015	0.25	0.013	-0.0023	-2.37	p<0.05
Idaea subsericeata	Satin Wave	380	334	-0.121	0.359	0.019	0.282	0.016	-0.0038	-3.10	p<0.05
Idaea sylvestraria	Dotted Border Wave	61	43	-0.295	0.316	0.042	0.206	0.032	-0.0054	-2.08	p<0.05
Idaea trigeminata	Treble Brown Spot	384	487	0.268	0.359	0.02	0.503	0.026	0.0070	4.39	p<0.05
Ipimorpha retusa	Double Kidney	144	142	-0.014	0.255	0.022	0.236	0.02	-0.0009	-0.64	NS
Ipimorpha subtusa	Olive	472	528	0.119	0.286	0.014	0.308	0.014	0.0011	1.11	NS
Itame brunneata	Rannoch Looper	25	45	0.800	0.147	0.03	0.253	0.038	0.0052	2.19	p<0.05
Jodia croceago	Orange Upperwing	17	1	-0.941	0.134	0.033	0.008	0.008	-0.0061	-3.71	p<0.05
Jodis lactearia	Little Emerald	539	427	-0.208	0.343	0.016	0.235	0.012	-0.0053	-5.40	p<0.05
Jordanita globulariae	Scarce Forester	16	7	-0.563	0.276	0.072	0.116	0.045	-0.0078	-1.88	NS
Lacanobia contigua	Beautiful Brocade	180	147	-0.183	0.254	0.019	0.183	0.015	-0.0035	-2.93	p<0.05
Lacanobia oleracea	Bright-line Brown-eye	1399	1494	0.068	0.946	0.035	0.998	0.037	0.0025	1.02	NS
Lacanobia suasa	Dog's Tooth	278	277	-0.004	0.284	0.018	0.264	0.016	-0.0010	-0.83	NS
Lacanobia thalassina	Pale-shouldered Brocade	1069	1034	-0.033	0.527	0.018	0.438	0.015	-0.0043	-3.80	p<0.05
Lacanobia w-latinum	Light Brocade	401	417	0.040	0.336	0.018	0.332	0.017	-0.0002	-0.16	NS
Lampropteryx otregiata	Devon Carpet	105	178	0.695	0.233	0.024	0.363	0.029	0.0063	3.45	p<0.05
Lampropteryx suffumata	Water Carpet	826	950	0.150	0.398	0.015	0.438	0.016	0.0020	1.82	NS
Laothoe populi	Poplar Hawk-moth	1391	1536	0.104	0.954	0.035	1.133	0.044	0.0087	3.18	p<0.05
Larentia clavaria	Mallow	397	339	-0.146	0.301	0.016	0.232	0.013	-0.0034	-3.35	p<0.05
Lasiocampa quercus	Oak Eggar	936	841	-0.101	0.526	0.019	0.392	0.015	-0.0065	-5.54	p<0.05
Lasiocampa trifolii	Grass Eggar	30	34	0.133	0.228	0.044	0.242	0.043	0.0007	0.23	NS
Laspeyria flexula	Beautiful Hook-tip	476	559	0.174	0.368	0.018	0.43	0.02	0.0030	2.30	p<0.05
Leucochlaena oditis	Beautiful Gothic	16	11	-0.313	0.507	0.135	0.286	0.091	-0.0108	-1.36	NS
Leucoma salicis	White Satin	494	425	-0.140	0.392	0.019	0.287	0.015	-0.0051	-4.34	p<0.05
Ligdia adustata	Scorched Carpet	542	615	0.135	0.412	0.02	0.449	0.02	0.0018	1.31	NS
Lithomoia solidaginis	Golden-rod Brindle	132	52	-0.606	0.254	0.023	0.09	0.013	-0.0080	-6.21	p<0.05
Lithophane hepatica	Pale Pinion	285	662	1.323	0.158	0.01	0.417	0.017	0.0126	13.13	p<0.05
Lithophane leautieri	Blair's Shoulder-knot	539	819	0.519	0.297	0.014	0.518	0.021	0.0108	8.76	p<0.05
Lithophane ornitopus	Grey Shoulder-knot	417	642	0.540	0.246	0.013	0.415	0.018	0.0082	7.61	p<0.05
Lithophane semibrunnea	Tawny Pinion	278	336	0.209	0.228	0.014	0.266	0.015	0.0019	1.85	NS
Lithosia quadra	Four-spotted Footman	95	239	1.516	0.137	0.014	0.346	0.024	0.0102	7.52	p<0.05
Lithostege griseata	Grey Carpet	20	35	0.750	0.221	0.052	0.386	0.073	0.0080	1.84	NS
Lobophora halterata	Seraphim	427	432	0.012	0.305	0.015	0.289	0.014	-0.0008	-0.78	NS
Lomaspilis marginata	Clouded Border	1369	1454	0.062	0.948	0.035	0.974	0.037	0.0013	0.51	NS
Lomographa bimaculata	White-pinion Spotted	572	715	0.250	0.327	0.015	0.406	0.017	0.0039	3.48	p<0.05
Lomographa temerata	Clouded Silver	1012	1211	0.197	0.591	0.022	0.765	0.029	0.0085	4.78	p<0.05

Luperina nickerlii	Sandhill Rustic	20	23	0.150	0.324	0.076	0.365	0.08	0.0020	0.37	NS
Luperina testacea	Flounced Rustic	1212	1233	0.017	0.718	0.026	0.657	0.023	-0.0030	-1.76	NS
Lycia hirtaria	Brindled Beauty	713	712	-0.001	0.433	0.018	0.37	0.015	-0.0031	-2.69	p<0.05
Lycia lapponaria	Rannoch Brindled Beauty	16	11	-0.313	0.226	0.061	0.135	0.041	-0.0044	-1.24	NS
Lycia zonaria	Belted Beauty	22	21	-0.045	0.408	0.092	0.293	0.079	-0.0056	-0.95	NS
Lycophotia porphyrea	True Lover's Knot	1158	1092	-0.057	0.717	0.025	0.562	0.02	-0.0076	-4.84	p<0.05
Lygephila pastinum	Blackneck	373	380	0.019	0.304	0.016	0.293	0.016	-0.0005	-0.49	NS
Lymantria dispar	Gypsy Moth	20	17	-0.150	0.262	0.061	0.233	0.057	-0.0014	-0.35	NS
Lymantria monacha	Black Arches	462	709	0.535	0.328	0.016	0.593	0.027	0.0129	8.44	p<0.05
Macaria alternata	Sharp-angled Peacock	258	383	0.484	0.382	0.026	0.549	0.033	0.0081	3.98	p<0.05
Macaria carbonaria	Netted Mountain Moth	22	17	-0.227	0.37	0.098	0.288	0.075	-0.0040	-0.66	NS
Macaria liturata	Tawny-barred Angle	894	879	-0.017	0.434	0.016	0.37	0.013	-0.0031	-3.10	p<0.05
Macaria notata	Peacock Moth	361	410	0.136	0.36	0.02	0.373	0.02	0.0006	0.46	NS
Macaria wauaria	V-Moth	488	82	-0.832	0.322	0.015	0.043	0.005	-0.0136	-17.65	p<0.05
Macrochilo cribrumalis	Dotted Fan-foot	91	97	0.066	0.344	0.039	0.355	0.039	0.0005	0.20	NS
Macroglossum stellatarum	Humming-bird Hawk-moth	753	1259	0.672	0.314	0.012	0.69	0.024	0.0183	14.01	p<0.05
Macrothylacia rubi	Fox Moth	728	631	-0.133	0.474	0.019	0.349	0.015	-0.0061	-5.16	p<0.05
Malacosoma castrensis	Ground Lackey	24	17	-0.292	0.451	0.103	0.268	0.069	-0.0089	-1.48	NS
Malacosoma neustria	Lackey	746	542	-0.273	0.705	0.032	0.322	0.015	-0.0187	-10.84	p<0.05
Mamestra brassicae	Cabbage Moth	1090	939	-0.139	0.597	0.021	0.401	0.014	-0.0096	-7.77	p<0.05
Meganola albula	Kent Black Arches	122	168	0.377	0.318	0.03	0.473	0.04	0.0076	3.10	p<0.05
Meganola strigula	Small Black Arches	30	17	-0.433	0.269	0.05	0.154	0.038	-0.0056	-1.83	NS
Melanchra persicariae	Dot Moth	995	990	-0.005	0.68	0.027	0.581	0.022	-0.0048	-2.84	p<0.05
Melanchra pisi	Broom Moth	1156	883	-0.236	0.631	0.022	0.34	0.012	-0.0142	-11.61	p<0.05
Melanthia procellata	Pretty Chalk Carpet	350	317	-0.094	0.383	0.022	0.289	0.017	-0.0046	-3.38	p<0.05
Menophra abruptaria	Waved Umber	644	770	0.196	0.415	0.018	0.508	0.021	0.0045	3.36	p<0.05
Mesoleuca albicillata	Beautiful Carpet	500	388	-0.224	0.306	0.014	0.203	0.01	-0.0050	-5.99	p<0.05
Mesoligia furuncula	Cloaked Minor	831	909	0.094	0.466	0.018	0.485	0.018	0.0009	0.75	NS
Mesoligia literosa	Rosy Minor	884	823	-0.069	0.462	0.017	0.37	0.014	-0.0045	-4.18	p<0.05
Miltochrista miniata	Rosy Footman	382	519	0.359	0.374	0.021	0.525	0.027	0.0074	4.41	p<0.05
Mimas tiliae	Lime Hawk-moth	651	838	0.287	0.406	0.018	0.551	0.023	0.0071	4.96	p<0.05
Minoa murinata	Drab Looper	62	45	-0.274	0.337	0.045	0.237	0.037	-0.0049	-1.72	NS
Moma alpium	Scarce Merveille du Jour	19	30	0.579	0.198	0.046	0.301	0.057	0.0050	1.41	NS
Mormo maura	Old Lady	598	688	0.151	0.298	0.013	0.334	0.013	0.0018	1.96	NS
Mythimna albipuncta	White-point	129	352	1.729	0.159	0.014	0.552	0.033	0.0192	10.96	p<0.05
Mythimna comma	Shoulder-striped Wainscot	960	934	-0.027	0.514	0.019	0.431	0.016	-0.0040	-3.34	p<0.05
Mythimna conigera	Brown-line Bright Eye	903	768	-0.150	0.479	0.018	0.339	0.013	-0.0068	-6.31	p<0.05
Mythimna favicolor	Mathew's Wainscot	50	47	-0.060	0.397	0.061	0.36	0.056	-0.0018	-0.45	NS
Mythimna ferrago	Clay	1241	1257	0.013	0.787	0.029	0.696	0.025	-0.0044	-2.38	p<0.05
Mythimna flammea	Flame Wainscot	53	37	-0.302	0.385	0.055	0.234	0.04	-0.0074	-2.22	p<0.05
Mythimna impura	Smoky Wainscot	1540	1592	0.034	1.275	0.05	1.232	0.049	-0.0021	-0.61	NS
Mythimna l-album	L-album Wainscot	88	165	0.875	0.27	0.03	0.509	0.045	0.0117	4.42	p<0.05
Mythimna litoralis	Shore Wainscot	81	62	-0.235	0.336	0.039	0.227	0.03	-0.0053	-2.22	p<0.05
Mythimna obsoleta	Obscure Wainscot	161	132	-0.180	0.327	0.027	0.238	0.021	-0.0043	-2.60	p<0.05
Mythimna pallens	Common Wainscot	1282	1351	0.054	0.803	0.029	0.799	0.029	-0.0002	-0.10	NS
Mythimna pudorina	Striped Wainscot	233	250	0.073	0.278	0.019	0.262	0.017	-0.0008	-0.63	NS
Mythimna putrescens	Devonshire Wainscot	27	31	0.148	0.34	0.068	0.265	0.05	-0.0037	-0.89	NS
Mythimna straminea	Southern Wainscot	334	376	0.126	0.317	0.018	0.334	0.018	0.0008	0.67	NS
Mythimna turca	Double Line	97	123	0.268	0.269	0.028	0.295	0.028	0.0013	0.66	NS
Mythimna unipuncta	White-speck	116	216	0.862	0.196	0.018	0.346	0.024	0.0073	5.00	p<0.05
Naenia typica	Gothic	763	714	-0.064	0.373	0.014	0.304	0.012	-0.0034	-3.74	p<0.05
Nebula salicata	Striped Twin-spot Carpet	205	92	-0.551	0.318	0.023	0.128	0.014	-0.0093	-7.06	p<0.05
Noctua comes	Lesser Yellow Underwing	1476	1569	0.063	1.106	0.042	1.177	0.046	0.0035	1.14	NS
Noctua fimbriata	Broad-bordered Yellow Underwing	988	1210	0.225	0.476	0.017	0.627	0.022	0.0074	5.43	p<0.05
Noctua interjecta	Least Yellow Underwing	855	971	0.136	0.504	0.02	0.565	0.022	0.0030	2.05	p<0.05
Noctua janthe	Lesser Broad-bordered Yellow Underwing	1419	1536	0.082	0.992	0.037	1.101	0.043	0.0053	1.92	NS
Noctua orbona	Lunar Yellow Underwing	112	55	-0.509	0.217	0.021	0.099	0.013	-0.0058	-4.78	p<0.05
Noctua pronuba	Large Yellow Underwing	1795	1865	0.039	2.528	0.113	2.934	0.146	0.0198	2.20	p<0.05
Nola confusalis	Least Black Arches	624	862	0.381	0.298	0.013	0.428	0.016	0.0063	6.31	p<0.05

Nola cucullatella	Short-cloaked Moth	769	708	-0.079	0.541	0.023	0.417	0.017	-0.0060	-4.34	p<0.05
Nonagria typhae	Bulrush Wainscot	571	629	0.102	0.312	0.014	0.326	0.014	0.0007	0.71	NS
Notodonta dromedarius	Iron Prominent	1087	1276	0.174	0.552	0.02	0.684	0.024	0.0064	4.23	p<0.05
Notodonta ziczac	Pebble Prominent	1142	1318	0.154	0.593	0.021	0.716	0.025	0.0060	3.77	p<0.05
Nudaria mundana	Muslin Footman	492	435	-0.116	0.388	0.019	0.304	0.015	-0.0041	-3.47	p<0.05
Nycteola revayana	Oak Nycteoline	410	632	0.541	0.227	0.012	0.367	0.016	0.0068	7.00	p<0.05
Ochropacha duplaris	Common Lutestring	779	842	0.081	0.38	0.015	0.38	0.014	0.0000	0.00	NS
Ochropleura plecta	Flame Shoulder	1526	1672	0.096	1.256	0.049	1.586	0.068	0.0161	3.94	p<0.05
Odezia atrata	Chimney Sweeper	664	537	-0.191	0.522	0.022	0.347	0.016	-0.0085	-6.43	p<0.05
Odontopera bidentata	Scalloped Hazel	1160	1223	0.054	0.626	0.022	0.605	0.021	-0.0010	-0.69	NS
Odontosia carmelita	Scarce Prominent	213	193	-0.094	0.291	0.021	0.253	0.019	-0.0019	-1.34	NS
Oligia fasciuncula	Middle-barred Minor	1285	1353	0.053	0.747	0.026	0.733	0.025	-0.0007	-0.39	NS
Oligia latruncula	Tawny Marbled Minor	753	693	-0.080	0.421	0.017	0.332	0.013	-0.0043	-4.16	p<0.05
Oligia strigilis	Marbled Minor	967	856	-0.115	0.499	0.018	0.361	0.013	-0.0067	-6.22	p<0.05
Oligia versicolor	Rufous Minor	559	604	0.081	0.347	0.015	0.346	0.015	0.0000	-0.05	NS
Omphaloscelis lunosa	Lunar Underwing	902	1052	0.166	0.467	0.017	0.558	0.02	0.0044	3.47	p<0.05
Operophtera brumata	Winter Moth	1072	956	-0.108	0.536	0.019	0.391	0.014	-0.0071	-6.14	p<0.05
Operophtera fagata	Northern Winter Moth	407	243	-0.403	0.303	0.016	0.167	0.011	-0.0066	-7.00	p<0.05
Opisthograptis luteolata	Brimstone Moth	1595	1691	0.060	1.536	0.063	1.71	0.076	0.0085	1.76	NS
Orgyia antiqua	Vapourer	820	882	0.076	0.409	0.016	0.414	0.015	0.0002	0.23	NS
Orgyia recens	Scarce Vapourer	15	8	-0.467	0.213	0.056	0.102	0.036	-0.0054	-1.67	NS
Oria musculosa	Brighton Wainscot	35	1	-0.971	0.3	0.053	0.007	0.007	-0.0143	-5.48	p<0.05
Orthonama vittata	Oblique Carpet	317	279	-0.120	0.255	0.015	0.199	0.012	-0.0027	-2.92	p<0.05
Orthosia cerasi	Common Quaker	1167	1385	0.187	0.631	0.022	0.835	0.03	0.0100	5.48	p<0.05
Orthosia cruda	Small Quaker	903	1085	0.202	0.446	0.017	0.546	0.019	0.0049	3.92	p<0.05
Orthosia gothica	Hebrew Character	1316	1507	0.145	0.783	0.028	0.994	0.037	0.0103	4.55	p<0.05
Orthosia gracilis	Powdered Quaker	830	856	0.031	0.389	0.015	0.363	0.013	-0.0013	-1.31	NS
Orthosia incerta	Clouded Drab	1143	1334	0.167	0.586	0.021	0.728	0.025	0.0069	4.35	p<0.05
Orthosia miniosa	Blossom Underwing	189	131	-0.307	0.243	0.018	0.162	0.014	-0.0040	-3.55	p<0.05
Orthosia munda	Twin-spotted Quaker	767	978	0.275	0.366	0.014	0.495	0.018	0.0063	5.66	p<0.05
Orthosia opima	Northern Drab	151	88	-0.417	0.215	0.018	0.118	0.013	-0.0047	-4.37	p<0.05
Orthosia populeti	Lead-coloured Drab	348	314	-0.098	0.297	0.016	0.253	0.015	-0.0021	-2.01	p<0.05
Ourapteryx sambucaria	Swallow-tailed Moth	1176	1216	0.034	0.777	0.029	0.739	0.027	-0.0019	-0.96	NS
Pachycnemia hippocastanaria	Horse Chestnut	80	76	-0.050	0.345	0.04	0.324	0.039	-0.0010	-0.38	NS
Panemeria tenebrata	Small Yellow Underwing	444	335	-0.245	0.3	0.015	0.202	0.011	-0.0048	-5.27	p<0.05
Panolis flammea	Pine Beauty	544	752	0.382	0.255	0.011	0.367	0.014	0.0055	6.29	p<0.05
Papestra biren	Glaucous Shears	237	193	-0.186	0.243	0.016	0.18	0.013	-0.0031	-3.06	p<0.05
Paracolax tristalis	Clay Fan-foot	29	14	-0.517	0.346	0.067	0.145	0.04	-0.0098	-2.58	p<0.05
Paradarisa consonaria	Square Spot	203	255	0.256	0.242	0.018	0.285	0.019	0.0021	1.64	NS
Paradrina clavipalpis	Pale Mottled Willow	990	977	-0.013	0.487	0.017	0.421	0.015	-0.0032	-2.91	p<0.05
Parascotia fuliginaria	Waved Black	126	159	0.262	0.281	0.026	0.378	0.032	0.0047	2.35	p<0.05
Parasemia plantaginis	Wood Tiger	326	189	-0.420	0.288	0.017	0.145	0.011	-0.0070	-7.06	p<0.05
Parastichtis suspecta	Suspected	390	327	-0.162	0.291	0.015	0.226	0.013	-0.0032	-3.27	p<0.05
Parastichtis ypsillon	Dingy Shears	525	527	0.004	0.318	0.015	0.295	0.013	-0.0011	-1.16	NS
Parectropis similaria	Brindled White-spot	246	279	0.134	0.31	0.021	0.349	0.022	0.0019	1.28	NS
Pasiphila chloerata	Sloe Pug	196	191	-0.026	0.301	0.022	0.285	0.021	-0.0008	-0.53	NS
Pasiphila debiliata	Bilberry Pug	49	53	0.082	0.233	0.034	0.242	0.034	0.0004	0.19	NS
Pasiphila rectangulata	Green Pug	975	1153	0.183	0.486	0.018	0.601	0.021	0.0056	4.16	p<0.05
Pechipogo strigilata	Common Fan-foot	80	17	-0.788	0.207	0.023	0.04	0.01	-0.0081	-6.66	p<0.05
Pelosia muscerda	Dotted Footman	18	32	0.778	0.21	0.051	0.361	0.066	0.0074	1.81	NS
Pelurga comitata	Dark Spinach	427	274	-0.358	0.343	0.017	0.186	0.011	-0.0077	-7.75	p<0.05
Perconia strigillaria	Grass Wave	137	107	-0.219	0.297	0.026	0.227	0.022	-0.0034	-2.06	p<0.05
Peribatodes rhomboidaria	Willow Beauty	1232	1326	0.076	0.803	0.03	0.837	0.031	0.0017	0.79	NS
Peridea anceps	Great Prominent	326	350	0.074	0.3	0.017	0.298	0.017	-0.0001	-0.08	NS
Perizoma affinitata	Rivulet	774	811	0.048	0.399	0.015	0.384	0.014	-0.0007	-0.73	NS
Perizoma albulata	Grass Rivulet	502	360	-0.283	0.306	0.014	0.195	0.01	-0.0054	-6.45	p<0.05
Perizoma alchemillata	Small Rivulet	1153	1176	0.020	0.62	0.022	0.564	0.019	-0.0027	-1.93	NS
Perizoma bifaciata	Barred Rivulet	358	307	-0.142	0.294	0.016	0.232	0.014	-0.0030	-2.92	p<0.05
Perizoma blandiata	Pretty Pinion	108	82	-0.241	0.286	0.028	0.175	0.02	-0.0054	-3.23	p<0.05

Perizoma didymata	Twin-spot Carpet	1139	842	-0.261	0.647	0.022	0.346	0.013	-0.0147	-11.78	p<0.05
Perizoma flavofasciata	Sandy Carpet	884	920	0.041	0.429	0.016	0.408	0.015	-0.0010	-0.96	NS
Perizoma minorata	Heath Rivulet	38	10	-0.737	0.235	0.039	0.052	0.017	-0.0089	-4.30	p<0.05
Perizoma sagittata	Marsh Carpet	22	8	-0.636	0.369	0.082	0.114	0.041	-0.0124	-2.78	p<0.05
Perizoma taeniata	Barred Carpet	29	32	0.103	0.173	0.032	0.173	0.031	0.0000	0.00	NS
Petrophora chlorosata	Brown Silver-line	1149	1292	0.124	0.625	0.022	0.702	0.025	0.0038	2.31	p<0.05
Phalera bucephala	Buff-tip	1202	1286	0.070	0.71	0.026	0.723	0.026	0.0006	0.35	NS
Pheosia gnoma	Lesser Swallow Prominent	1145	1268	0.107	0.612	0.022	0.67	0.023	0.0028	1.82	NS
Pheosia tremula	Swallow Prominent	976	1161	0.190	0.48	0.017	0.593	0.021	0.0055	4.18	p<0.05
Phibalapteryx virgata	Oblique Striped	61	35	-0.426	0.266	0.035	0.145	0.025	-0.0059	-2.81	p<0.05
Phigalia pilosaria	Pale Brindled Beauty	778	810	0.041	0.36	0.014	0.35	0.013	-0.0005	-0.52	NS
Philereme transversata	Dark Umber	317	340	0.073	0.345	0.021	0.346	0.02	0.0000	0.03	NS
Philereme vetulata	Brown Scallop	233	200	-0.142	0.326	0.023	0.254	0.019	-0.0035	-2.41	p<0.05
Phlogophora meticulosa	Angle Shades	1363	1537	0.128	0.876	0.032	1.103	0.042	0.0111	4.30	p<0.05
Photedes captiuncula	Least Minor	16	4	-0.750	0.216	0.057	0.054	0.027	-0.0079	-2.57	p<0.05
Photedes minima	Small Dotted Buff	1024	942	-0.080	0.516	0.018	0.397	0.014	-0.0058	-5.22	p<0.05
Phragmatobia fuliginosa	Ruby Tiger	1150	1327	0.154	0.614	0.022	0.739	0.026	0.0061	3.67	p<0.05
Phytometra viridaria	Small Purple-barred	310	218	-0.297	0.269	0.016	0.169	0.012	-0.0049	-5.00	p<0.05
Plagodis dolabraria	Scorched Wing	788	1005	0.275	0.428	0.017	0.57	0.021	0.0069	5.26	p<0.05
Plagodis pulveraria	Barred Umber	340	287	-0.156	0.281	0.016	0.207	0.012	-0.0036	-3.70	p<0.05
Plemyria rubiginata	Blue-bordered Carpet	623	643	0.032	0.312	0.013	0.3	0.012	-0.0006	-0.68	NS
Plusia festucae	Gold Spot	742	971	0.309	0.349	0.014	0.472	0.017	0.0060	5.59	p<0.05
Plusia putnami	Lempke's Gold Spot	164	247	0.506	0.234	0.019	0.36	0.024	0.0061	4.12	p<0.05
Poecilocampa populi	December Moth	802	793	-0.011	0.376	0.014	0.33	0.012	-0.0022	-2.49	p<0.05
Polia bombycina	Pale Shining Brown	161	15	-0.907	0.265	0.021	0.02	0.005	-0.0120	-11.35	p<0.05
Polia nebulosa	Grey Arches	730	687	-0.059	0.391	0.016	0.315	0.013	-0.0037	-3.69	p<0.05
Polia trimaculosa	Silvery Arches	91	25	-0.725	0.239	0.026	0.058	0.012	-0.0088	-6.32	p<0.05
Polychrysia moneta	Golden Plusia	471	242	-0.486	0.334	0.016	0.141	0.009	-0.0094	-10.51	p<0.05
Polymixis flavicincta	Large Ranunculus	303	379	0.251	0.293	0.018	0.338	0.018	0.0022	1.77	NS
Polymixis lichenea	Feathered Ranunculus	290	302	0.041	0.403	0.025	0.369	0.023	-0.0017	-1.00	NS
Polymixis xanthomista	Black-banded	22	23	0.045	0.332	0.075	0.222	0.049	-0.0054	-1.23	NS
Polyploca ridens	Frosted Green	360	422	0.172	0.294	0.016	0.336	0.017	0.0020	1.80	NS
Protodeltote pygarga	Marbled White Spot	397	682	0.718	0.266	0.014	0.532	0.023	0.0130	9.88	p<0.05
Protolampra sobrina	Cousin German	31	25	-0.194	0.378	0.073	0.237	0.05	-0.0069	-1.59	NS
Pseudoips prasinana	Green Silver-lines	730	916	0.255	0.353	0.014	0.451	0.017	0.0048	4.45	p<0.05
Pseudopanthera macularia	Speckled Yellow	422	346	-0.180	0.366	0.019	0.258	0.014	-0.0053	-4.58	p<0.05
Pseudoterpna pruinata	Grass Emerald	603	386	-0.360	0.403	0.018	0.211	0.011	-0.0094	-9.10	p<0.05
Pterapherapteryx sexalata	Small Seraphim	302	327	0.083	0.303	0.018	0.294	0.017	-0.0004	-0.36	NS
Pterostoma palpina	Pale Prominent	990	1140	0.152	0.55	0.02	0.64	0.023	0.0044	2.95	p<0.05
Ptilodon capucina	Coxcomb Prominent	1274	1334	0.047	0.783	0.028	0.755	0.027	-0.0014	-0.72	NS
Ptilodon cucullina	Maple Prominent	227	281	0.238	0.41	0.03	0.551	0.039	0.0069	2.87	p<0.05
Ptilophora plumigera	Plumed Prominent	22	15	-0.318	0.263	0.057	0.171	0.045	-0.0045	-1.27	NS
Pyrrhia umbra	Bordered Sallow	332	284	-0.145	0.299	0.017	0.234	0.014	-0.0032	-2.95	p<0.05
Rheumaptera cervinalis	Scarce Tissue	152	114	-0.250	0.247	0.021	0.172	0.016	-0.0037	-2.84	p<0.05
Rheumaptera hastata	Argent & Sable	155	111	-0.284	0.213	0.018	0.132	0.014	-0.0040	-3.55	p<0.05
Rheumaptera undulata	Scallop Shell	420	452	0.076	0.293	0.015	0.29	0.014	-0.0001	-0.15	NS
Rhizedra lutosa	Large Wainscot	563	642	0.140	0.303	0.013	0.328	0.014	0.0012	1.31	NS
Rhyacia simulans	Dotted Rustic	393	108	-0.725	0.43	0.023	0.095	0.009	-0.0163	-13.56	p<0.05
Rivula sericealis	Straw Dot	853	1466	0.719	0.364	0.014	1.046	0.04	0.0333	16.09	p<0.05
Rusina ferruginea	Brown Rustic	1150	1114	-0.031	0.628	0.022	0.514	0.018	-0.0056	-4.01	p<0.05
Saturnia pavonia	Emperor Moth	747	595	-0.203	0.47	0.019	0.308	0.013	-0.0079	-7.04	p<0.05
Schrankia costaestrigalis	Pinion-streaked Snout	351	623	0.775	0.205	0.011	0.379	0.016	0.0085	8.96	p<0.05
Schrankia taenialis	White-line Snout	83	83	0.000	0.278	0.031	0.23	0.026	-0.0023	-1.19	NS
Scoliopteryx libatrix	Herald	1090	1129	0.036	0.548	0.019	0.512	0.018	-0.0018	-1.38	NS
Scopula emutaria	Rosy Wave	75	69	-0.080	0.389	0.047	0.347	0.044	-0.0020	-0.65	NS
Scopula floslactata	Cream Wave	625	632	0.011	0.327	0.014	0.306	0.013	-0.0010	-1.10	NS
Scopula imitaria	Small Blood-vein	696	794	0.141	0.492	0.021	0.557	0.023	0.0032	2.09	p<0.05
Scopula immutata	Lesser Cream Wave	342	349	0.020	0.29	0.016	0.273	0.015	-0.0008	-0.78	NS
Scopula marginepunctata	Mullein Wave	273	291	0.066	0.406	0.026	0.399	0.025	-0.0003	-0.19	NS

Scopula ornata	Lace Border	22	17	-0.227	0.18	0.039	0.135	0.033	-0.0022	-0.88	NS
Scopula rubiginata	Tawny Wave	27	37	0.370	0.252	0.05	0.321	0.055	0.0034	0.93	NS
Scopula ternata	Smoky Wave	212	195	-0.080	0.296	0.021	0.245	0.018	-0.0025	-1.84	NS
Scotopteryx bipunctaria	Chalk Carpet	137	93	-0.321	0.303	0.027	0.187	0.02	-0.0057	-3.45	p<0.05
Scotopteryx chenopodiata	Shaded Broad-bar	1300	1200	-0.077	0.839	0.031	0.596	0.021	-0.0119	-6.49	p<0.05
Scotopteryx luridata	July Belle	453	280	-0.382	0.349	0.017	0.181	0.011	-0.0082	-8.30	p<0.05
Scotopteryx mucronata	Lead Belle	234	168	-0.282	0.277	0.019	0.174	0.014	-0.0050	-4.36	p<0.05
Selenia dentaria	Early Thorn	1313	1391	0.059	0.853	0.031	0.849	0.031	-0.0002	-0.09	NS
Selenia lunularia	Lunar Thorn	490	304	-0.380	0.323	0.015	0.167	0.01	-0.0076	-8.65	p<0.05
Selenia tetralunaria	Purple Thorn	837	982	0.173	0.452	0.017	0.52	0.019	0.0033	2.67	p<0.05
Selidosema brunnearia	Bordered Grey	42	18	-0.571	0.348	0.055	0.148	0.035	-0.0098	-3.07	p<0.05
Semiaspilates ochrearia	Yellow Belle	163	199	0.221	0.332	0.028	0.39	0.03	0.0028	1.41	NS
Sesia bembeciformis	Lunar Hornet Moth	210	152	-0.276	0.24	0.017	0.164	0.013	-0.0037	-3.55	p<0.05
Setina irrorella	Dew Moth	27	14	-0.481	0.33	0.065	0.163	0.045	-0.0081	-2.11	p<0.05
Shargacucullia lychnitis	Striped Lychnis	37	36	-0.027	0.334	0.059	0.319	0.056	-0.0007	-0.18	NS
Shargacucullia verbasci	Mullein	580	563	-0.029	0.378	0.017	0.315	0.014	-0.0031	-2.86	p<0.05
Sideridis albicolon	White Colon	107	85	-0.206	0.324	0.032	0.238	0.026	-0.0042	-2.09	p<0.05
Simyra albovenosa	Reed Dagger	62	90	0.452	0.264	0.035	0.381	0.043	0.0057	2.11	p<0.05
Smerinthus ocellata	Eyed Hawk-moth	761	821	0.079	0.443	0.018	0.446	0.018	0.0001	0.12	NS
Spaelotis ravida	Stout Dart	224	9	-0.960	0.371	0.026	0.012	0.004	-0.0175	-13.65	p<0.05
Spargania luctuata	White-banded Carpet	26	24	-0.077	0.308	0.064	0.232	0.05	-0.0037	-0.94	NS
Sphinx ligustri	Privet Hawk-moth	522	599	0.148	0.411	0.02	0.434	0.02	0.0011	0.81	NS
Spilosoma lubricipeda	White Ermine	1420	1505	0.060	1.026	0.039	1.047	0.04	0.0010	0.38	NS
Spilosoma luteum	Buff Ermine	1211	1321	0.091	0.849	0.032	0.97	0.039	0.0059	2.40	p<0.05
Spilosoma urticae	Water Ermine	62	55	-0.113	0.236	0.031	0.193	0.027	-0.0021	-1.05	NS
Standfussiana lucernea	Northern Rustic	166	112	-0.325	0.307	0.025	0.193	0.019	-0.0056	-3.63	p<0.05
Stauropus fagi	Lobster Moth	438	547	0.249	0.369	0.019	0.461	0.022	0.0045	3.16	p<0.05
Stilbia anomala	Anomalous	349	218	-0.375	0.383	0.021	0.197	0.014	-0.0091	-7.37	p<0.05
Syngrapha interrogationis	Scarce Silver Y	199	187	-0.060	0.256	0.019	0.223	0.017	-0.0016	-1.29	NS
Tethea ocularis	Figure of Eighty	700	829	0.184	0.427	0.018	0.509	0.02	0.0040	3.05	p<0.05
Tethea or	Poplar Lutestring	211	196	-0.071	0.263	0.019	0.224	0.016	-0.0019	-1.57	NS
Tetheella fluctuosa	Satin Lutestring	128	143	0.117	0.306	0.028	0.336	0.03	0.0015	0.73	NS
Thalpophila matura	Straw Underwing	776	654	-0.157	0.519	0.021	0.361	0.015	-0.0077	-6.12	p<0.05
Thera britannica	Spruce Carpet	675	1127	0.670	0.284	0.011	0.596	0.021	0.0152	13.16	p<0.05
Thera cognata	Chestnut-coloured Carpet	66	52	-0.212	0.226	0.029	0.156	0.022	-0.0034	-1.92	NS
Thera cupressata	Cypress Carpet	32	139	3.344	0.114	0.021	0.622	0.058	0.0248	8.24	p<0.05
Thera firmata	Pine Carpet	513	629	0.226	0.284	0.013	0.352	0.015	0.0033	3.43	p<0.05
Thera juniperata	Juniper Carpet	273	284	0.040	0.281	0.018	0.283	0.017	0.0001	0.08	NS
Thera obeliscata	Grey Pine Carpet	1098	1144	0.042	0.556	0.019	0.528	0.018	-0.0014	-1.07	NS
Theria primaria	Early Moth	685	508	-0.258	0.378	0.015	0.227	0.01	-0.0074	-8.38	p<0.05
Tholera cespitis	Hedge Rustic	564	374	-0.337	0.329	0.014	0.185	0.01	-0.0070	-8.37	p<0.05
Tholera decimalis	Feathered Gothic	781	602	-0.229	0.43	0.017	0.264	0.011	-0.0081	-8.20	p<0.05
Thumatha senex	Round-winged Muslin	378	368	-0.026	0.333	0.018	0.306	0.017	-0.0013	-1.09	NS
Thyatira batis	Peach Blossom	1060	1137	0.073	0.562	0.02	0.566	0.02	0.0002	0.14	NS
Timandra comae	Blood-vein	915	1049	0.146	0.628	0.025	0.758	0.031	0.0063	3.26	p<0.05
Trichiura crataegi	Pale Eggar	547	344	-0.371	0.373	0.017	0.194	0.011	-0.0087	-8.84	p<0.05
Trichopteryx carpinata	Early Tooth-striped	594	731	0.231	0.309	0.013	0.374	0.015	0.0032	3.27	p<0.05
Trichopteryx polycommata	Barred Tooth-striped	44	32	-0.273	0.235	0.036	0.147	0.027	-0.0043	-1.96	NS
Triphosa dubitata	Tissue	251	146	-0.418	0.214	0.014	0.113	0.009	-0.0049	-6.07	p<0.05
Trisateles emortualis	Olive Crescent	10	22	1.200	0.168	0.054	0.404	0.089	0.0115	2.27	p<0.05
Tyria jacobaeae	Cinnabar	1138	1263	0.110	0.743	0.028	0.834	0.032	0.0044	2.14	p<0.05
Tyta luctuosa	Four-spotted	42	37	-0.119	0.13	0.02	0.107	0.018	-0.0011	-0.85	NS
Venusia cambrica	Welsh Wave	250	236	-0.056	0.3	0.02	0.259	0.017	-0.0020	-1.56	NS
Watsonalla binaria	Oak Hook-tip	740	851	0.150	0.466	0.019	0.534	0.021	0.0033	2.40	p<0.05
Watsonalla cultraria	Barred Hook-tip	340	368	0.082	0.305	0.017	0.308	0.017	0.0001	0.12	NS
Xanthia aurago	Barred Sallow	488	661	0.355	0.312	0.015	0.453	0.02	0.0069	5.64	p<0.05
Xanthia citrago	Orange Sallow	371	455	0.226	0.238	0.013	0.284	0.014	0.0022	2.41	p<0.05
Xanthia gilvago	Dusky-lemon Sallow	359	238	-0.337	0.264	0.014	0.159	0.011	-0.0051	-5.90	p<0.05
Xanthia icteritia	Sallow	1042	1019	-0.022	0.505	0.018	0.436	0.015	-0.0034	-2.94	p<0.05

Xanthia ocellaris	Pale-lemon Sallow	54	32	-0.407	0.325	0.045	0.186	0.033	-0.0068	-2.49	p<0.05
Xanthia togata	Pink-barred Sallow	914	996	0.090	0.405	0.015	0.416	0.014	0.0005	0.54	NS
Xanthorhoe biriviata	Balsam Carpet	52	67	0.288	0.276	0.039	0.335	0.042	0.0029	1.03	NS
Xanthorhoe decoloraria	Red Carpet	263	158	-0.399	0.434	0.028	0.229	0.019	-0.0100	-6.06	p<0.05
Xanthorhoe designata	Flame Carpet	1013	1312	0.295	0.501	0.018	0.753	0.026	0.0123	7.97	p<0.05
Xanthorhoe ferrugata	Dark-barred Twin-spot Carpet	1023	858	-0.161	0.596	0.022	0.375	0.014	-0.0108	-8.47	p<0.05
Xanthorhoe fluctuata	Garden Carpet	1449	1461	0.008	1.038	0.039	0.915	0.033	-0.0060	-2.41	p<0.05
Xanthorhoe montanata	Silver-ground Carpet	1719	1720	0.001	1.953	0.083	1.679	0.073	-0.0134	-2.48	p<0.05
Xanthorhoe quadrifasiata	Large Twin-spot Carpet	346	435	0.257	0.346	0.02	0.44	0.024	0.0046	3.01	p<0.05
Xanthorhoe spadicearia	Red Twin-spot Carpet	965	1039	0.077	0.508	0.019	0.506	0.018	-0.0001	-0.08	NS
Xestia agathina	Heath Rustic	376	396	0.053	0.286	0.015	0.286	0.015	0.0000	0.00	NS
Xestia alpicola	Northern Dart	15	11	-0.267	0.163	0.044	0.12	0.037	-0.0021	-0.75	NS
Xestia ashworthii	Ashworth's Rustic	26	11	-0.577	0.387	0.084	0.191	0.061	-0.0096	-1.89	NS
Xestia baja	Dotted Clay	993	845	-0.149	0.554	0.02	0.379	0.014	-0.0085	-7.17	p<0.05
Xestia castanea	Neglected Rustic	314	263	-0.162	0.278	0.016	0.213	0.013	-0.0032	-3.15	p<0.05
Xestia c-nigrum	Setaceous Hebrew Character	1240	1387	0.119	0.715	0.025	0.843	0.03	0.0062	3.28	p<0.05
Xestia ditrapezium	Triple-spotted Clay	411	390	-0.051	0.323	0.017	0.274	0.014	-0.0024	-2.22	p<0.05
Xestia rhomboidea	Square-spotted Clay	135	155	0.148	0.193	0.017	0.215	0.018	0.0011	0.89	NS
Xestia sexstrigata	Six-striped Rustic	1081	1103	0.020	0.539	0.019	0.491	0.017	-0.0023	-1.88	NS
Xestia triangulum	Double Square-spot	1251	1382	0.105	0.737	0.026	0.822	0.03	0.0041	2.14	p<0.05
Xestia xanthographa	Square-spot Rustic	1418	1526	0.076	0.959	0.035	1.041	0.039	0.0040	1.56	NS
Xylena exsoleta	Sword-grass	118	47	-0.602	0.153	0.014	0.06	0.009	-0.0045	-5.59	p<0.05
Xylena vetusta	Red Sword-grass	286	445	0.556	0.191	0.012	0.293	0.014	0.0050	5.53	p<0.05
Xylocampa areola	Early Grey	909	1118	0.230	0.464	0.017	0.591	0.021	0.0062	4.70	p<0.05
Zanclognatha tarsipennalis	Fan-foot	874	1119	0.280	0.456	0.017	0.669	0.025	0.0104	7.05	p<0.05
Zeuzera pyrina	Leopard Moth	566	638	0.127	0.392	0.018	0.432	0.019	0.0020	1.53	NS
Zygaena filipendulae	Six-spot Burnet	972	1016	0.045	0.511	0.019	0.474	0.017	-0.0018	-1.45	NS
Zygaena lonicerae	Narrow-bordered Five-spot Burnet	580	527	-0.091	0.414	0.019	0.343	0.016	-0.0035	-2.86	p<0.05
Zygaena purpuralis	Transparent Burnet	19	13	-0.316	0.733	0.196	0.397	0.129	-0.0164	-1.43	NS
Zygaena trifolii	Five-spot Burnet	341	191	-0.440	0.345	0.02	0.147	0.011	-0.0097	-8.67	p<0.05

* GB macro-moths were classified into Resident, Resident+immigrant and Non-resident species. The latter group (i.e. those occuring in GB **only** as immigrant, vagrant, adventive and non-breeding species) were excluded from the study. Resident+immigrant species maintain year-round, breeding populations in parts of GB but also occur as immigrants.
